# Estimating Metabolic Fluxes Using a Maximum Network Flexibility Paradigm

**DOI:** 10.1371/journal.pone.0139665

**Published:** 2015-10-12

**Authors:** Wout Megchelenbrink, Sergio Rossell, Martijn A. Huynen, Richard A. Notebaart, Elena Marchiori

**Affiliations:** 1 Institute for Computing and Information Sciences (ICIS), Radboud University, Nijmegen, the Netherlands; 2 Centre for Molecular and Biomolecular Informatics (CMBI), Radboud University Medical Centre, Nijmegen, the Netherlands; 3 Centre for Systems Biology and Bioenergetics (CSBB), Radboud University Medical Centre, Nijmegen, the Netherlands; 4 Netherlands Cancer Institute (NKI), Amsterdam, the Netherlands; Tata Institute of Fundamental Research, INDIA

## Abstract

**Motivation:**

Genome-scale metabolic networks can be modeled in a constraint-based fashion. Reaction stoichiometry combined with flux capacity constraints determine the space of allowable reaction rates. This space is often large and a central challenge in metabolic modeling is finding the biologically most relevant flux distributions. A widely used method is flux balance analysis (FBA), which optimizes a biologically relevant objective such as growth or ATP production. Although FBA has proven to be highly useful for predicting growth and byproduct secretion, it cannot predict the intracellular fluxes under all environmental conditions. Therefore, alternative strategies have been developed to select flux distributions that are in agreement with experimental “omics” data, or by incorporating experimental flux measurements. The latter, unfortunately can only be applied to a limited set of reactions and is currently not feasible at the genome-scale. On the other hand, it has been observed that micro-organisms favor a suboptimal growth rate, possibly in exchange for a more “flexible” metabolic network. Instead of dedicating the internal network state to an optimal growth rate in one condition, a suboptimal growth rate is used, that allows for an easier switch to other nutrient sources. A small decrease in growth rate is exchanged for a relatively large gain in metabolic capability to adapt to changing environmental conditions.

**Results:**

Here, we propose Maximum Metabolic Flexibility (MMF) a computational method that utilizes this observation to find the most probable intracellular flux distributions. By mapping measured flux data from central metabolism to the genome-scale models of *Escherichia coli* and *Saccharomyces cerevisiae* we show that i) indeed, most of the measured fluxes agree with a high adaptability of the network, ii) this result can be used to further reduce the space of feasible solutions iii) this reduced space improves the quantitative predictions made by FBA and contains a significantly larger fraction of the measured fluxes compared to the flux space that was reduced by a uniform sampling approach and iv) MMF can be used to select reactions in the network that contribute most to the steady-state flux space. Constraining the selected reactions improves the quantitative predictions of FBA considerably more than adding an equal amount of flux constraints, selected using a more naïve approach. Our method can be applied to any cell type without requiring prior information.

**Availability:**

MMF is freely available as a MATLAB plugin at: http://cs.ru.nl/~wmegchel/mmf.

## Introduction

Advances in obtaining quantitative “omics” data have led to the availability of genome-scale metabolic network reconstructions for many organisms. Successful metabolic modelling examples range from predicting the impact of cell perturbation experiments in micro-organisms [1] and *in silico* yield optimization of valuable products such as bioethanol [2] to metabolic engineering for drug synthesis [3] and tumor vulnerability studies in cancer cells [4–8].

At the heart of these models lies the stoichiometric matrix (**S**), containing *m* metabolites and *n* reactions. Entry S_i,j_ denotes the stoichiometric coefficient of metabolite *i* in reaction *j*. The allowable flux range v_j_ for reaction *j* is bounded by the mass-balance equations (considered at steady-state) and flux capacity constraints

$$\frac{\text{d}\vec{\text{x}}}{\text{dt}}=\text{S}\vec{\text{v}}=0$$ (1)

$${\text{v}}_{\text{j}}^{\text{lb}}\leq {\text{v}}_{\text{j}}\leq {\text{v}}_{\text{j}}^{\text{ub}},\forall \text{j}\in \{1,2,\ldots ,\text{n}\}$$ (2)

where $\vec{\text{x}}$ and $\vec{\text{v}}$ are vectors denoting the metabolite concentrations and reaction rates respectively. In metabolic networks the reactions typically outnumber the metabolites, leaving the system of linear equations **S** underdetermined [9]. This means that there is no unique solution, but rather a convex space of (infinitely many) feasible flux distributions [10], known as the steady-state solution space. Knowledge of the actual flux distribution the organism utilizes is of great importance for many biological engineering purposes [9,11], making reduction of the solution space a central problem in metabolic modeling. Since the reaction stoichiometry in eq (1) is fixed, reduction of the solution space can only be achieved by tightening the feasible flux ranges in eq (2). Methods for reducing the feasible fluxes to those that are biologically most relevant can be divided into three main categories.

i) Computational methods that select flux distributions based on optimization of a biologically sound objective, such as biomass or ATP yield. Flux Balance Analysis (FBA) [10,12] is arguably the most applied technique that has shown to be accurate in predicting maximum growth [13] and byproduct secretion rates [14] for micro-organisms. Often, the flux distribution obtained by FBA is not unique and multiple optima exist. Flux Variability Analysis (FVA) [15] can be viewed as an extension of FBA that, instead of finding a unique flux distribution, computes the minimum and maximum allowable flux through each reaction while optimizing an objective function. To further reduce the space of alternative optimal solutions, variants of FBA are used. A method that is often applied is parsimonious enzyme usage (pFBA)[16]. This technique selects among the optimal flux distributions the one that minimizes the sum of absolute fluxes, using the rationale that a cell minimizes its enzymatic cost when alternative optimal flux paths exist. Other methods, tailored to minimize the enzymatic cost exist [17–20], but require organism specific parameters that are not widely available, such as metabolite and enzyme concentrations or the Gibbs free energy change associated with each reaction.

ii) The most reliable method is direct measurement of the unknown intra- and extracellular fluxes. Extracellular or boundary reactions such as glucose and oxygen consumption, together with growth rates and byproduct secretion rates such as acetate, ethanol and CO_2_ are measured on a routinely basis. Unfortunately, measuring intracellular fluxes is currently limited by the available experimental techniques. A successful technique that measures intracellular fluxes is metabolic flux analysis (MFA) [21–23]. MFA uses isotopic (13C) labeling combined with a computational approach to uniquely identify the reaction rates inside central (carbon) metabolism. A drawback of the MFA method is that it is currently mainly limited to central metabolism and can therefore not be applied on the genome-scale.

iii) Computational methods that use other omics sources such as gene- or protein expression [24–28]. The basic idea behind most of these methods is to maximize the agreement between high (low) expression and active (inactive) pathways. Although the relation between gene-expression and metabolic flux is not straightforward and fluxes may not correlate well with the expression of their enzyme-coding genes [29,30], this is a method that can be applied on the genome-scale. Many methods have shown to be highly predictive in a qualitative sense (predicting active versus inactive fluxes), but a recent review revealed that most are not well-suited for making quantitative flux predictions [31].

It has been shown that microbes that normally grow in various environments favor a suboptimal growth in order to keep a metabolic state that allows for an easier switch towards other nutrient conditions [32,33]. Here, we utilize this tradeoff between adaptability and growth to define a novel algorithm that allows for selecting flux ranges from the suboptimal growth space that correspond to maximum metabolic flexibility (MMF) in the network.

We evaluated our method on the genome-scale models of *E*. *coli* and *S*. *cerevisiae* and show that measured reaction rates indeed correspond to a high MMF value within the solution space corresponding to suboptimal growth. This information can be used to discard fluxes that would severely affect the organisms’ metabolic flexibility and effectively reduces the flux range for each reaction considered. We compared our method with a uniform sampling approach and show that MMF provides a smaller prediction error rate when flux ranges are reduced at a similar cutoff. By applying pFBA after discarding all flux ranges that violate the MMF paradigm by more than 5%, better quantitative flux estimates are obtained. Finally, we demonstrate that our method selects reactions that contribute substantially to the uncertainty in the network. Measurement of the fluxes selected by our method provides a larger reduction and subsequently better prediction than can be expected from an approach that cannot estimate these most likely fluxes.

## Materials and Methods

### Computing the total flux range (TFR) distribution

Our method uses the idea that micro-organisms, which can grow in various environments typically grow at a suboptimal rate on a certain substrate, to allow for an easier adaptation towards other nutrient sources [32,33]. Our hypothesis is therefore that the routing of metabolic fluxes in these micro-organism is such that they facilitate a substantial growth rate and are furthermore tuned to a maximal metabolic “flexibility”. To clarify this, we introduce a measure called the total flux range (TFR). The TFR is the sum of the feasible flux ranges for a set of *n* reactions:$\sum_{i=1}^{n}(v_{i}^{ub}-v_{i}^{lb})$ and can easily be computed using FVA. The *n* reactions can be composed to cover all metabolic reactions in the network or only a specific subset of interest, such as the TCA cycle or the glycolysis pathway. The method proceeds in two phases. First, the feasible flux range $\left(v_{i}^{ub}-v_{i}^{lb}\right)$ for each reaction *i* is binned into *b* bins. Denote with *v*_*i*_ = *δ*_*j*_ that the lower- and upper flux bound for reaction *i* are constrained to the lower- and upper bound of bin *j*. TFR(v|*v*_*i*_ = *δ*_*j*_) then denotes the total flux range given the tightened bound on reaction *i*. TFR(v|*v*_*i*_ = *δ*_*j*_) is computed by adjusting the lower- and upper bound of reaction *i* and propagating this constraint through the other reactions in the network (e.g. using FVA). Finally, this expression is normalized, such that TFR(v|*v*_*i*_ = *δ*_*j*_) denotes the TFR fraction remaining after constraining reaction *i*.

**Fig 1:**
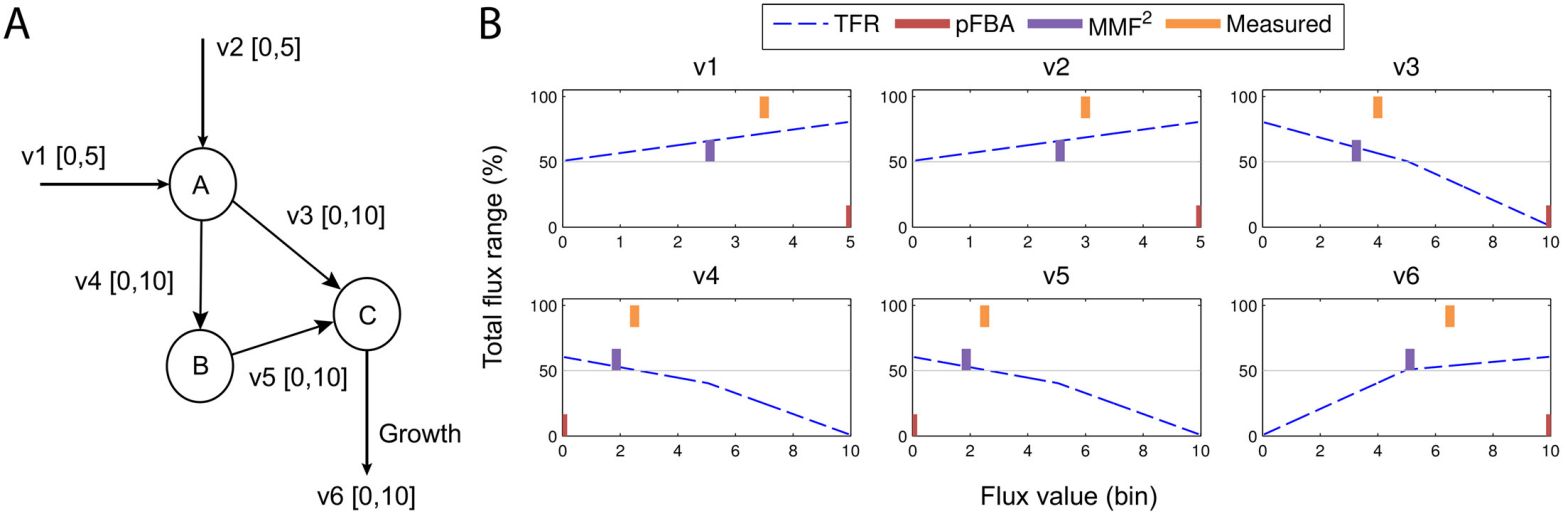
A toy example of the MMS method. (A) A toy network with 3 metabolites, connected by 6 reactions. Flux lower- and upper bounds are denoted between brackets. (B) The TFR distribution (relative to the original TFR) for each reaction.

In the second phase, a unique flux distribution can be chosen with respect to the maximum metabolic flexibility paradigm. A first and straightforward approach is to discard all flux values that cause the TFR to drop below a certain cutoff. This can then be used to further constrain the flux bounds, such that the space of possible flux distributions shrinks. This approach is illustrated for the toy network in Fig 1a. The network consists of three metabolites connected by six reactions. Reaction v1 and v2 produce metabolite A, which is converted into biomass either through intermediate metabolite B or C. Fig 1b illustrates the TFR distribution (purple) for each reaction as a function of the constrained flux through that reaction (here, for simplicity we set the lower- and upper flux bound to the center of the bin). Notice that for instance for the uptake reactions v1 and v2 a higher uptake rate corresponds to a higher TFR. This occurs because reaction v3 and v4 can obtain a wide range of fluxes given enough input through either v1 or v2. Similarly, when reaction v3 obtains the highest rate of 10 mmol/gDW/h, there is only one feasible flux distribution and therefore TFR(v|v_3_ = [10,10]) is 0. Notice that when the TFR cutoff is set to 0.5, the maximum flux through reaction v3 is approximately 5.0 mmol/gDW/h and the minimal flux through the growth reaction (v6) should also be 5.0. Using this approach, we can exclude flux distributions that significantly violate the metabolic flexibility principle. To obtain a unique flux distribution, we can apply for instance pFBA to the network where the flux bounds were constrained such that they only allow flux distributions that satisfy a predefined minimal flexibility.

### Computing the flux distribution with maximum metabolic flexibility (MMF)

A second option is to choose the flux distribution that maximizes the TFR under the mass-balance and flux capacity constraints directly. This can be useful in the case that a clear biological objective such as growth is not present, which is the case for instance in most mammalian cells. Because the TFR distribution was computed for each reaction separately, selecting for each reaction the flux value that maximizes the TFR yields a non-steady-state flux distribution $\hat{p}$. This can be solved by selecting the steady-state flux distribution that minimizes the overall Euclidean distance to the mode (peak) of each of the binned flux ranges. However, for some reactions, the TFR is hardly sensitive to the chosen flux value (a uniform distribution), whereas for others there is a clear mode with maximum TFR (S1 File). The peaked distributions provide a better estimate of the real flux an organism can obtain and we therefore assign more weight to these reactions. The flux distribution that globally maximizes the TFR can be found by solving the following constrained weighted least-squares problem:

$$\begin{array}{l} {\text{min}}_{v}||Wv-W\hat{p}|{|}_{2}^{2}\\ s.t.\\ Sv=0\\ v_{lb,i}\leq v_{i}\leq v_{ub,i},\forall i \end{array}$$ (3)

Here, W is a diagonal matrix of weights and $\hat{p}$ is the (unconstrained) flux distribution that maximizes the TFR. The weight of a flux reflects how well it can be predicted with the MMF method. Therefore, distributions with a clear mode should receive more weight than uniform distributions. Shannon’s information entropy is used to determine the weight of each reaction *i*:

$$W_{i}={\text{log}}_{2}(b)-\sum_{\text{j=1}}^{\text{b}}\text{T}{\text{FR}}_{\text{i,j}}{\text{log}}_{2}{\text{(TFR}}_{\text{i,j}}\text{)}$$ (4)

Note that when the TFR_i_ has maximum entropy (uniform distribution), the weight *i* is minimal (0). The vector of weights $\vec{W}$ is normalized such that all entries sum up to 1. Eq (3) can be formulated as a quadratic program that finds the flux distribution such that the network “flexibility” is maximized under the imposed constraints.

### Using the TFR distribution to select flux measurements

MMF can be used to select or prioritize flux measurements. If measured flux data is available, it can be used to reduce the flux capacity constraints (the lower- and upper bounds). Furthermore, propagation (using FVA) of the new bounds typically reduces the bounds of other reactions as well. The amount of reduction caused depends mainly on three factors. First, the reduction of the measured reaction depends on the experimental precision. More importantly, how this reduction affects the feasible ranges of other reactions in the network depends secondly on what the actual flux is that was measured and furthermore on how that reaction is connected to the rest of the network (the network topology). For instance, if a high excretion rate of ethanol was measured, then much of the carbon excreted cannot go to other pathways (under the steady-state assumption). On the other hand, when a low ethanol excretion flux is detected, then it is still unknown how for instance carbon is excreted in order to satisfy the steady-state constraint. In other words, the organism might excrete other products such as acetate, glycerol and lactate or dedicate much of this flux to growth. Thus, a high excretion rate would typically provide a much larger reduction of the feasible space than a low excretion rate.

The TFR distribution is helpful, because it provides a reasonable estimate of the flux through a reaction (S1 File). Furthermore, we can immediately see how much reduction of the TFR can be obtained at each possible measurement outcome. Using the maximum flexibility principle, fluxes are selected where the mode of the TFR distribution is a global minimum. Any measurement outcome will reduce the TFR to at least this value. When the measured flux deviates strongly from the expected value, the TFR reduction will be even stronger.

### Metabolic models and flux data

A recent review about computational methods that integrate quantitative “omics” (gene-expression) data, showed that pFBA in general provides better quantitative flux estimates than most computational methods that integrate this omics data [31]. Although pFBA predicts some fluxes with high accuracy, predicted rates for other reactions can be distant from the rates that have been experimentally determined. We demonstrate the applicability of our method using the same genome-scale metabolic models and experimentally verified fluxes as in [31]. In particular, we used the Escherichia coli iAF1260 [34] and the Saccharomyces cerevisiae iMM904 [35] network reconstructions. The yeast iTO977 network [36] used in [31] was replaced by the iMM904 model, because the former did not yield a feasible growth rate when FBA was applied. For the E. coli model, fluxes have been measured in batch culture by Holm et al. [37] and in a chemostat environment by Ishii et al. [38]. The reaction rates for S. cerevisiae have been measured at varying oxygen consumption rates by Rintala and coworkers [39].

## Results

### The total flux range of genome-scale metabolic models

Feasible flux distributions form a high dimensional convex steady-state solution space. The hypervolume of this space indicates how “many” (since the space is continuous, there is actually an infinite number of solutions) alternative solutions exist. Unfortunately, exact computation of this volume is infeasible for genome-scale models [40,41] and reliable estimation of this volume is extremely hard [42]. Summing the feasible ranges over all reactions is a somewhat crude, but useful approximation.

**Fig 2:**
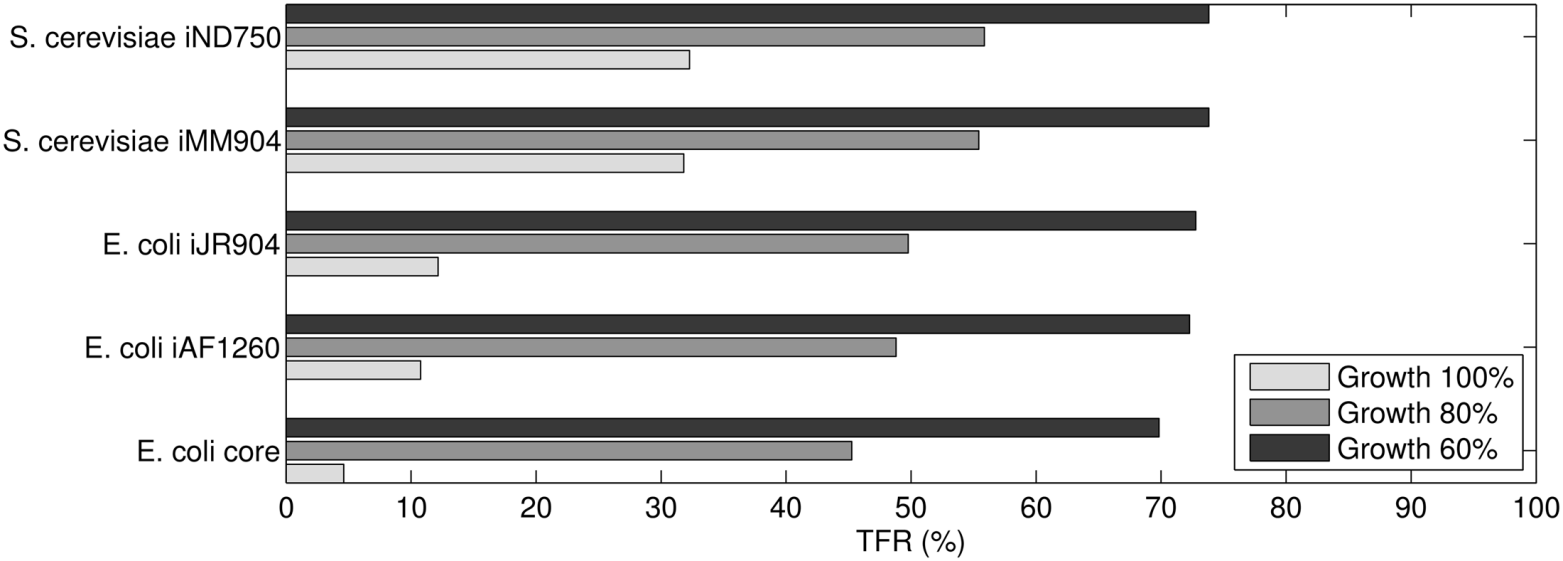
TFR for various genome-scale metabolic reconstructions of E. coli and S. cerevisiae. The TFR was computed while obtaining a minimum growth output of 60, 80 or 100 percent of the maximum value computed by FBA.

It is known that often, organisms do not grow at the theoretical optimal rate computed by FBA [43], but at a suboptimal rate. This is especially the case when nutrients are available in excess or the available oxygen is limited. The exact deviation between the predicted and actual growth rate depends on the experimental conditions. For the E. coli and yeast models we considered, the measured growth rates are between 60% and 80% of the theoretical optimum computed by FBA. Fig 2 shows that under this suboptimal growth, the space of alternative flux distributions is considerably larger than when only distributions satisfying optimal growth are considered. The hypothesis that metabolism in micro-organisms optimizes for growth allows FBA to effectively reduce the feasible flux ranges to only 10% to 30% compared to those in a minimal glucose medium without any maximization of growth. However, when the maximum biomass output is constrained to the measured suboptimal growth rate, this reduction is considerably less. Between 50% to even 70% of the original flux ranges (without optimizing for growth) satisfy the measured suboptimal growth rate. For organisms growing in a complex medium, this reduction is even less and thus a large space of flux distributions that agree with the measured growth rate exist. Thus, there is need to further narrow down this space of suboptimal growth solutions, using a secondary objective. We applied MMF, to maximize the flexibility, or adjustment capability of the metabolic network.

### Reducing the total flux range

**Fig 3:**
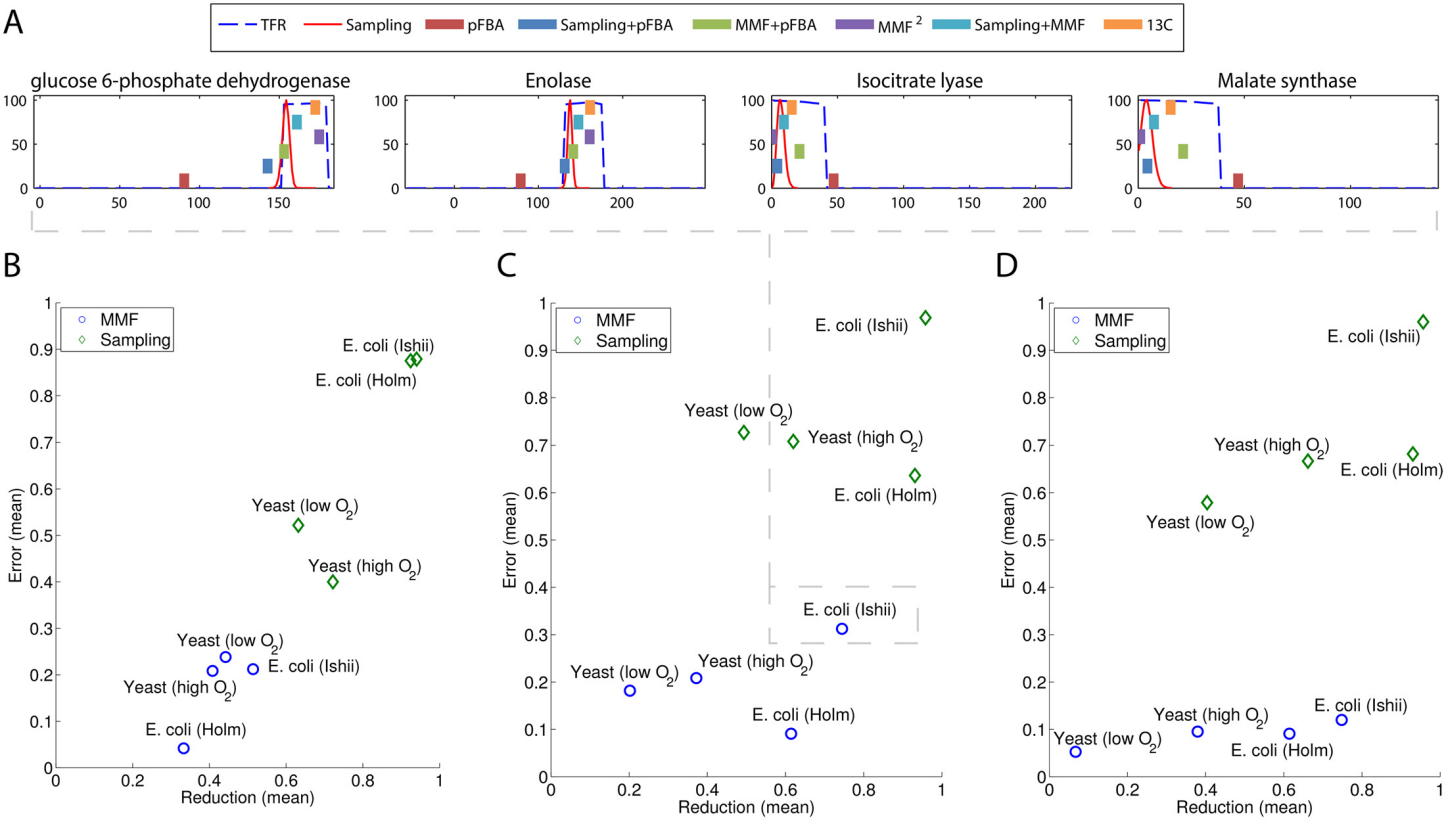
Illustration of the purpose of MMF on four reaction in central carbon metabolism. (A) The fluxes estimated by pFBA (red) are distant from the measured rates and are also outside the 0.95 TFR range. After applying MMF, the fluxes estimated by pFBA are much closer to the measured rates. Notice that the measured rates are within the 0.95 TFR range, but are generally not captured by the ACHR sampling distribution. (B-D) TFR reduction vs error in scenario 1–3 respectively. The MMF method provides less reduction of the flux ranges, but also has a considerably smaller error rate. The MMF performs best, when the growth rate (scenario 2) and the key exchange fluxes (scenario 3) are constrained.

By reducing the feasible ranges of the fluxes, better estimates can be made on how flux is actually distributed through the cell. A computational approach suitable for reducing the space of (suboptimal) feasible solutions is uniform random sampling [44–46]. By random sampling the feasible space, empirical probability density functions (pdf) can be defined for every reaction in the network. These can then be used to tighten the lower- and upper flux bounds of each reaction by discarding flux values with very low probability. The MMF distribution can be used in a similar fashion by excluding reaction rates that cause the TFR to drop below a certain cutoff. Fig 3a illustrates this procedure, for four reactions in the E. coli genome-scale model (for the other reactions and networks see S1 File). The sampling distributions (red lines) are narrow, meaning that many feasible flux values are actually never sampled and are therefore unlikely to happen given the network stoichiometry and flux capacity constraints. We also considered the MMF distribution and discarded all flux values that caused the TFR to drop below 0.95 (dashed blue lines). Although flux ranges based on the TFR distributions are wider, in contrast to the sampling procedure, they often include the flux rates measured by 13C MFA (orange squares).

In general, it is desirable to reduce the feasible space without excluding the real (i.e. the measured) fluxes. Fig 3b–3d compares the reduction of the solution space with the fraction of measured fluxes that are outside the reduced space; the mean error. In particular, Fig 3b shows these results for scenario 1, where only the glucose and oxygen uptake rates were constrained. In Fig 3c, the biomass flux was additionally constrained (scenario 2) and in Fig 3d all measured exchange reactions were constrained (scenario 3). The same trend is observed as in Fig 3a; while the sampling method obtains a large reduction of the space, it also excludes most of the measured fluxes leading to a high error rate. At a TFR cutoff of 0.95, the MMF method is more conservative compared to sampling. A smaller reduction of the space is obtained, but most of the measured reaction rates remain in the reduced space. When the cutoff is increased to 0.99, the TFR reduction becomes similar to that achieved by the sampling method (S2 File). Notice that, especially in the scenarios for which MMF was designed (finding flux distributions in the suboptimal space, i.e. where the growth rate is known), it achieves a smaller error rate than the sampling approach (Fig 3c–3d and Figs 2–3). In the remainder of this paper, we favor the smaller error over a large reduction and use the cutoff of 0.95.

### Quantitative flux estimation

A reduced solution space still does not provide a unique flux distribution. However, this can be obtained using either MMF to find the flux distribution that globally maximizes the TFR or by using pFBA on a model with refined bounds. Fig 3a illustrates how the predictions made by pFBA improve when the MMF method was used to preprocess the model and narrow the flux bounds. In this particular example, E. coli was grown in chemostat culture at a dilution rate of 0.2/h [38] and the model’s glucose and oxygen uptake as well as the biomass production rates were constrained to those experimentally measured (scenario 2). In this case, pFBA underestimates some of the major fluxes in glycolysis (fluxes catalyzed by GAPD and enolase) and overestimates some of the reactions in the TCA cycle (ICT lyase and malate synthase). By first tightening the flux lower- and upper bounds, such that the TFR of each reaction is above 0.95 and then rerunning pFBA, better estimates of the fluxes through these reactions (green squares) are obtained. Similarly, by first applying MMF to find the bounds that retain at least 0.95 of the original TFR, and then applying the global MMF optimization procedure (MMF^2^) in eqs 3 and 4, we obtain a flux distribution that is closer to the measured values than those computed using pFBA alone.

**Fig 4:**
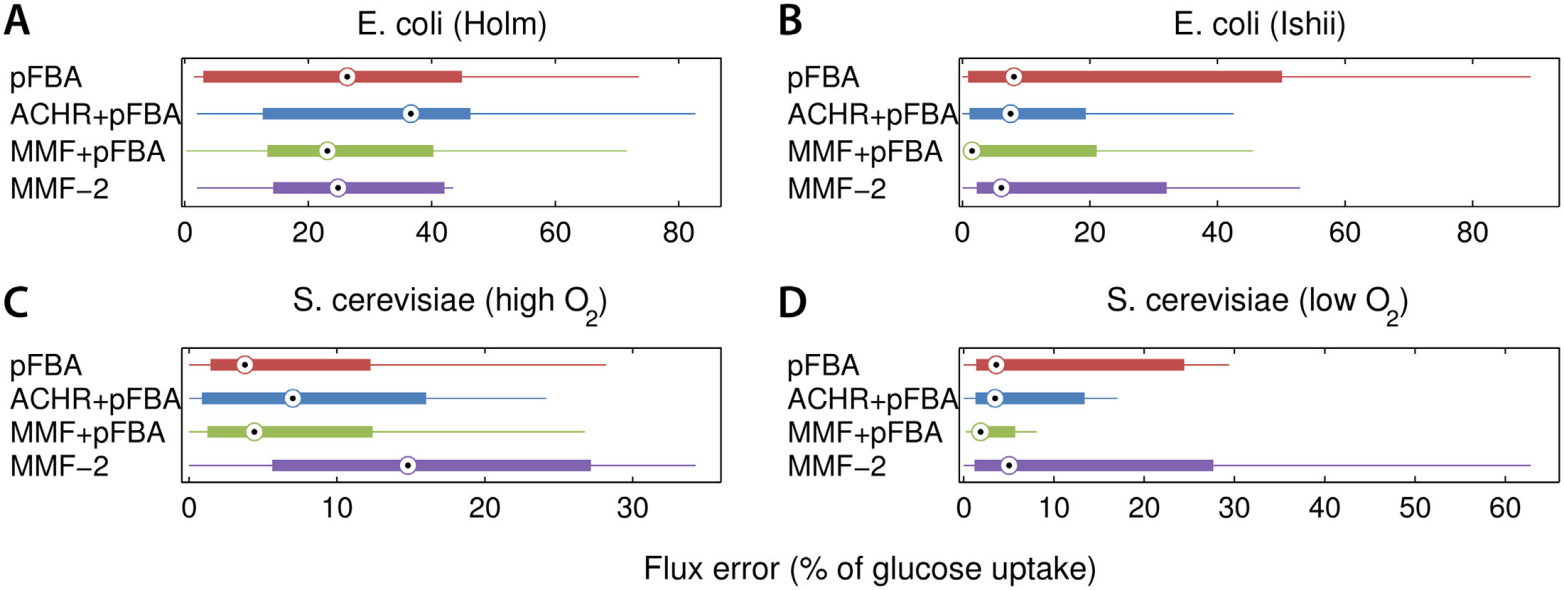
Flux prediction error for scenario 2. By first applying MMF, the predictions made by pFBA (MMF + pFBA) improve. The error improves in particular, when the biomass optimization paradigm may not hold (b and d). Furthermore, global optimization of the flexibility works pretty well, except for yeast growing under high oxygen conditions. In this case, yeast produces much biomass and fluxes estimated by pFBA are more accurate. By first applying ACHR, the feasible flux ranges are pruned to heavily, leading to worse flux estimates.

Fig 4 shows the mean error for the aforementioned models in scenario 2 (see S3 File for all conditions). Notice that the quantitative fluxes estimated by pFBA improve when MMF was applied in advance to refine the flux ranges. Again, this holds in particular for the chemostat models, where the maximum growth assumption used by pFBA may not hold due to limited oxygen availability (Fig 4b and 4d). In the absence of oxygen, yeast uses the fermentation pathways to produce biomass and excretes ethanol at a high rate. It is known that the flux predictions made by FBA are incorrect in this case [43] and refining the flux bounds with MMF helps to “guide” pFBA to a more accurate flux distribution. Notice that the MMF^2^ method works reasonably well for most models, except for S. cerevisiae growing under aerobic (high oxygen) conditions. The pFBA method, which assumes a maximization of the biomass yield is clearly a better approach here. Although MMF^2^ does not outperform MMF+pFBA or pFBA alone, it can be used to estimate fluxes for models where an optimization objective such a as growth is not applicable.

### Selecting flux measurements that optimally reduce the solution space

We applied the MMF method to select among the measured fluxes, those that are expected to maximally reduce the TFR. Selecting fluxes that have a large impact on the TFR may help to reduce valuable experimental time and cost. We iteratively selected one reaction and constrained the model with the measured flux until seven fluxes were selected. Because adding an extra constraint always reduces the TFR, we compared our method with two simple approaches. A rather naïve approach is to select reactions randomly from those that have available measurement data. The second approach always selects those reactions that have the largest distance between their upper- and lower bound (called the *MaxSpan* reactions). Although the flux range of these *MaxSpan* reactions can be reduced considerably, they may or may not have a large constraining effect on other flux ranges in the network.

**Fig 5:**
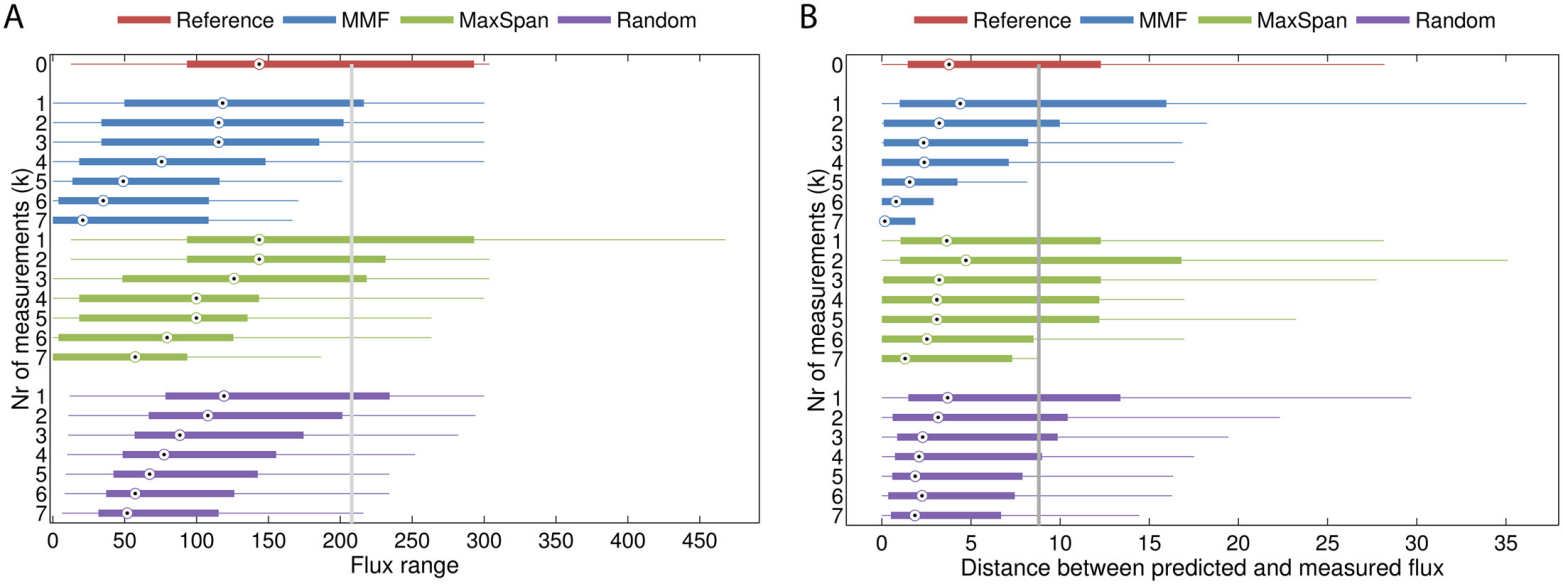
TFR reduction and pFBA improvement of MMF compared to random and “MaxSpan” selection on the S. cerevisiae iMM904 model (high oxygen). (A) The MMF method selects flux measurements that provide a larger reduction of the flux ranges compared to the MaxSpan or random measurements. (B) Fluxes predicted by pFBA obtain the smallest errors when the reactions selected by MMF are measured.

**Fig 6:**
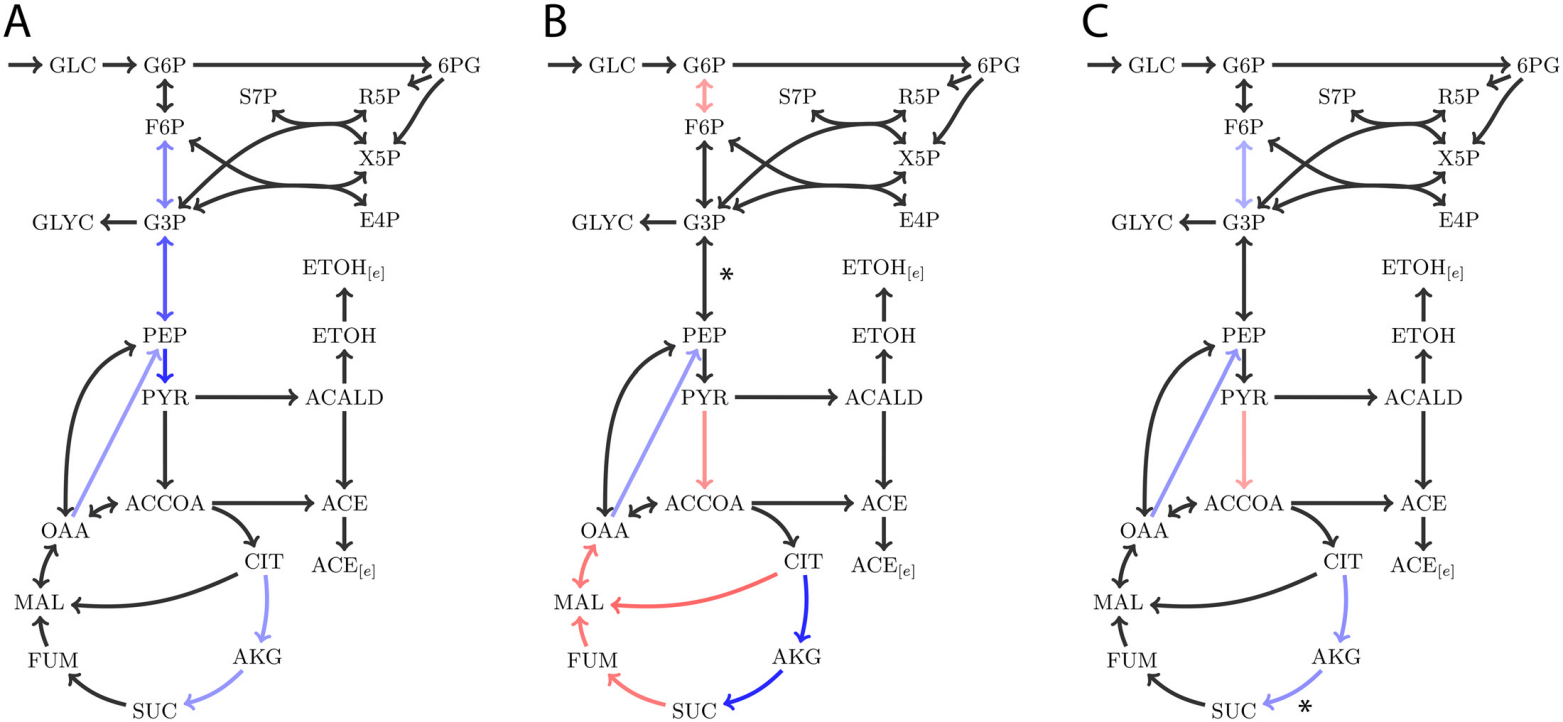
Effect of flux selection by MMF for explained with a network reconstruction of S. cerevisiae central metabolism. Red arrows denote overestimated fluxes by pFBA, compared to the measured data. Blue arrows denote underestimated fluxes. Using subsequent measurements in glycolysis (g3p -> pep) and the TCA cycle (succinyl-coA -> succinate), the pFBA estimates are much closer to the measured fluxes.

As a proof of concept, we looked specifically for reduction of the flux ranges for reactions inside central metabolism. Since flux data for other pathways was not available, little reduction can be expected within these pathways. Fig 5a shows that indeed, the flux ranges in the S. cerevisiae model are reduced faster when reactions selected by MMF are constrained with measured fluxes compared to the other methods (for all models and scenarios see S4 File). Fig 5b shows that, as expected, the larger reduction obtained after measurement of these reactions also results in better prediction of the fluxes by pFBA (see S5 File for all models and scenarios). Notice that although the TFR decreases with each flux measurement, this does not guarantee a better prediction of pFBA. The reason that the error can increase is that the additional constraint causes pFBA to perform a major flux rerouting, which is actually a worse estimate than the flux routing before imposing the extra constraint. Fig 6 illustrates this behavior for a subset of the reactions from the genome-scale model of S. cerevisiae (those residing in central metabolism). Some reactions in the glycolysis pathway have a large flux range and pFBA actually underestimates the flux through these reactions (Fig 6a). MMF selects the reaction G3P -> PEP for measurement. Due to the increased flux through glycolysis, more pyruvate enters the mitochondria. Apparently, the minimal sum of fluxes constraint drives a large portion of the flux through a shortcut which converts citrate into malate. As a consequence, reactions in the right part of the TCA cycle have underestimated rates and the fluxes in the left part are overestimated. A second measurement constrains the flux from succinyl-coa to succinate, which results in a much more accurate prediction.

## Discussion and Conclusion

A major advantage of constraint-based metabolic modeling is that relatively few constraints are required to predict quantitative traits such as a cell’s maximum growth rate, or uptake and secretion rates of key metabolites. The main drawback is, that the underdetermined nature of these networks, combined with a limited set of available constraints makes it hard to compute or estimate many flux distributions accurately. Computational methods such as FBA and pFBA have been successfully applied to find the flux distributions that correspond with a maximization of the growth rate. However, micro-organisms rarely grow at this theoretical maximum rate, but–depending on the available substrates and species- at rates typically between 60%-90% of this optimum. Importantly, even a small relaxation of the growth rate from 100% to 90% of the maximum computed by FBA, increases the amount of alternative optimal flux distributions considerably. The FBA solution to a system that is constrained to have a maximum of say, 90% depends mainly on the linear programming algorithm used and has no biological relevance compared to any other flux distribution within this suboptimal space. Using pFBA improves the situation, because the shortest absolute flux path is chosen from the optimal alternatives and thus thermodynamically infeasible cycles are avoided. The MMF method can be viewed as a (computationally expensive) alternative to minimizing the absolute sum of fluxes. Instead, a flux path is chosen that maximizes the “flexibility” or robustness of the network towards varying conditions, while maintaining a (sub)optimal growth rate. This robustness is believed to be an evolutionary design principle of the metabolic networks of many organisms and protects the cell against internal defects and varying environmental conditions [47,48]. The TFR summarizes the allowable flux ranges and thus can be viewed as a measure of metabolic robustness. We rarely observed flux distributions that severely limit the TFR. By using this simple observation, we were able to refine the alternative suboptimal solution space found by FVA, by redefining flux ranges such that they satisfy a predefined TFR (here 0.95 times the original TFR). Applying FBA or pFBA to this redefined space prevents that a flux route is selected that severely limits the metabolic robustness of the organism.

We would like to discuss our comparison with the uniform sampling approach. First, uniform sampling of metabolic networks is often done by the ACHR [49] method; a method that–contrary to the original Hit-and-Run method [50]- has no theoretical guarantees to converge towards a uniform distribution. The applicability of the ACHR method for large-scale metabolic networks has recently been challenged [51,52] and it is questionable to what extent the flux distributions sampled from the steady-state solution space are truly a uniform random sample. On the other hand, binning and propagating the flux values as done in MMF, has some resemblance with belief propagation; a method that has also been applied to metabolic networks [42,53]. Constraining the flux of one reaction and propagating this constraint through the network is exactly what belief propagation does. Then, the flux distribution with maximum TFR is a proxy to the one with maximum posterior belief. Thus, whether the reduced error rate of MMF compared to sampling is really due to the optimization of “metabolic flexibility” or is mainly due to the limitations of the ACHR method remains unclear.

Our method can also be used on networks that are made tissue- or environment specific with computational methods that integrate omics data with genome-scale networks, such as iMAT [25], GIM3E [54] or EXAMO [26]. Despite the significant reduction that is often obtained by these methods, a large space of solutions remains.

MMF can also be used to identify the key intracellular reactions. Here, to test the performance of our method, we used flux data obtained with MFA, since data obtained with other techniques is scarce at the moment. Before MMF would be applicable as a measurement selection tool for MFA techniques, the main obstacle that has to be solved is taking into account the intrinsic coupling that exist between the tracers used and the pathways that are measured. That is, reactions measured with MFA cannot be chosen one at a time and the choice of isotopic label determines which reaction rates will be measured. A possible solution would be to extend the algorithm to a greedy heuristic that tries to select multiple consecutive measurements that best partition the flux space into smaller subspaces. This would be useful to compute a priori which labeling scheme provides most information about the network under study, especially since the error rates can also be computed in advance [55]. Our method can also be applied in association with Kinetic Flux Profiling (KFP) [56]. KFP is a mass spectrometry based approach to measure individual fluxes through metabolic networks by pulsechase feeding of heavy isotope (13C, 15N) labeled nutrients, and does not require specific assumptions about the network.

Taken together, we have presented a novel method, which predicts metabolic fluxes by adopting a maximum network flexibility paradigm. Our method can be used to further narrow the solution space in genome-scale metabolic networks and thereby improve existing methods such as pFBA. Our method can predict (intra)cellular fluxes in the absence of known cellular objectives and can be used to find important hubs in the metabolic network that contribute most to the large range of alternative distributions.

## Supporting Information

S1 File Metabolic flux distribution estimated by ACHR sampling and by the proposed MMF method for scenario 2. In this scenario, the glucose and oxygen consumption rates were set to their measured values, and the biomass flux was constrained to the observed growth rate. The TFR distribution depicts for each reaction the feasible rate that allows a TFR ≥ 0.95. Notice that most measured fluxes are indeed within the range that allows for this large “network flexibility”. The narrow sampling distributions allow for a larger reduction of the flux space, but unfortunately often do not capture the measured (13C) flux. Rectangular shapes indicate the predicted (pFBA and MMF variants) and measured flux (13C). *E*. *coli iAF1260* (Holm et al.) network **(Fig A)**. *E*. *coli iAF1260* (Ishii et al.) network **(Fig B)**. *S*. *cerevisiae iMM904* high O2 (Rintala et al.) network **(Fig C)**. *S*. *cerevisiae iMM904* low O2 (Rintala et al.) network **(Fig D)**.
(PDF)

S2 File Tradeoff between reduction of the flux ranges and the number of measured fluxes outside the reduced space (error rate). ACHR sampling compared to the MMF approach with TFR ≥ 0.99. Scenario 1: glucose and oxygen uptakes are constrained. MMF seems to perform better for the E. coli models compared to uniform sampling. For the yeast model with low oxygen, the reduction achieved by MMF is larger, but the error is also larger. Finally, uniform sampling performs better for the high oxygen yeast model. Notice that since the growth rate was not constrained, MMF in fact optimizes for “network flexibility” as its *primary* objective, which is probably incorrect **(Fig A)**. Scenario 2: the uptake rates of glucose and oxygen, as well as the measured growth rate are set in the model. MMF performs better than random sampling for at least 3 of the 4 models (**Fig B)**. Scenario 3: all exchange rates are constrained. Again MMF performs better on at least 3 of the 4 models **(Fig C)**.
(PDF)

S3 File Flux prediction error by pFBA, pFBA after applying ACHR sampling and pFBA after applying our MMF method. Scenario 1: constrained glucose and oxygen consumption. MMF does not consider biomass pressure, but is designed to optimize the network flexibility under sub optimal growth rates. Therefore, it does not perform well in this scenario, where the growth rate is unknown **(Fig A)**. Scenario 2: the growth rate is constrained in addition to the glucose and oxygen consumption rates. In this scenario MMF performs better, because a sub optimal solution space is considered **(Fig B)**. Scenario 3: all measured exchange fluxes are constrained. In this case, the MMF paradigm is also valid (**Fig C)**.
(PDF)

S4 File TFR reduction in as a function of the number of measurements. The grey line denotes the mean flux range in the reference model. Scenario 1: fixed glucose and oxygen uptake rates. In this setting, MMF provides the best reduction of the flux ranges for all models **(Fig A)**. Scenario 2: the uptake rates of glucose and oxygen, as well as the measured growth rate are set in the model. Again MMF provides the best reduction of the flux ranges for all models **(Fig B)**. Scenario 3: all exchange rates are constrained. Also in this case, the best overall TFR reduction is achieved by MMF **(Fig C)**.
(PDF)

S5 File pFBA prediction error as a function of the number of measurements. The grey line denotes the mean error in the reference model. Scenario 1: constrained glucose and oxygen rates. A smaller TFR does not necessarily correlate with a reduced pFBA error **(Fig A)**. Scenario 2: the glucose and oxygen rates, as well as the biomass production rate are constrained. In most models, the strongly reduced prediction error correlates well with the reduced TFR, except for the E. coli iAF1260 (Ishii et al.) model **(Fig B)**. Scenario 3: all exchange fluxes are constrained. Again, the error does not monotonically decrease with the number of constrained reactions, but does converge towards zero **(Fig C)**.
(PDF)
