# Supplementary figures and images for: Estimating Metabolic Fluxes Using a Maximum Network Flexibility Paradigm
Source: PLoS One. 2015 Oct 12;10(10):e0139665. doi: 10.1371/journal.pone.0139665 (PMC4601694; doi:10.1371/journal.pone.0139665)

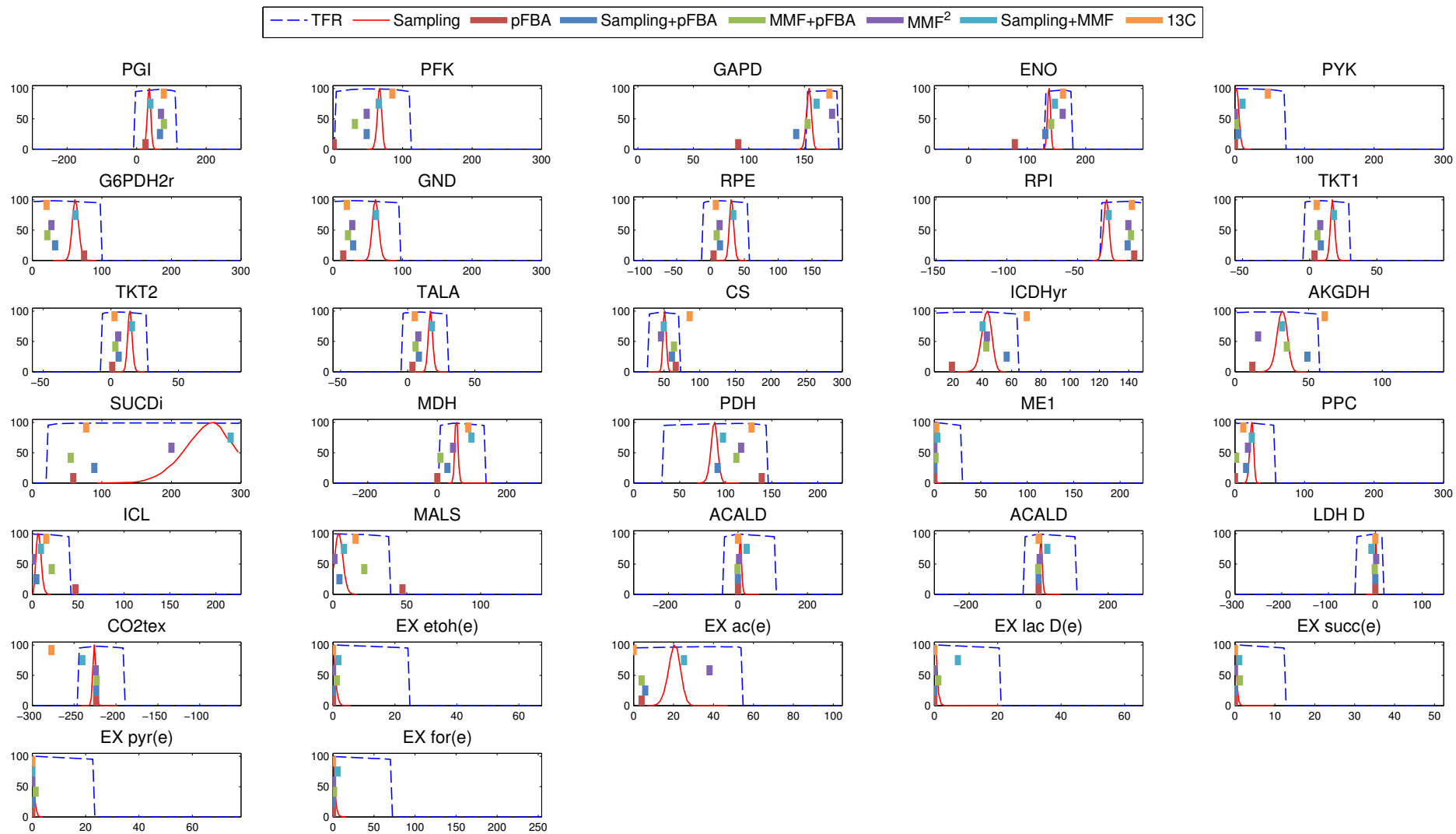

Figure 1

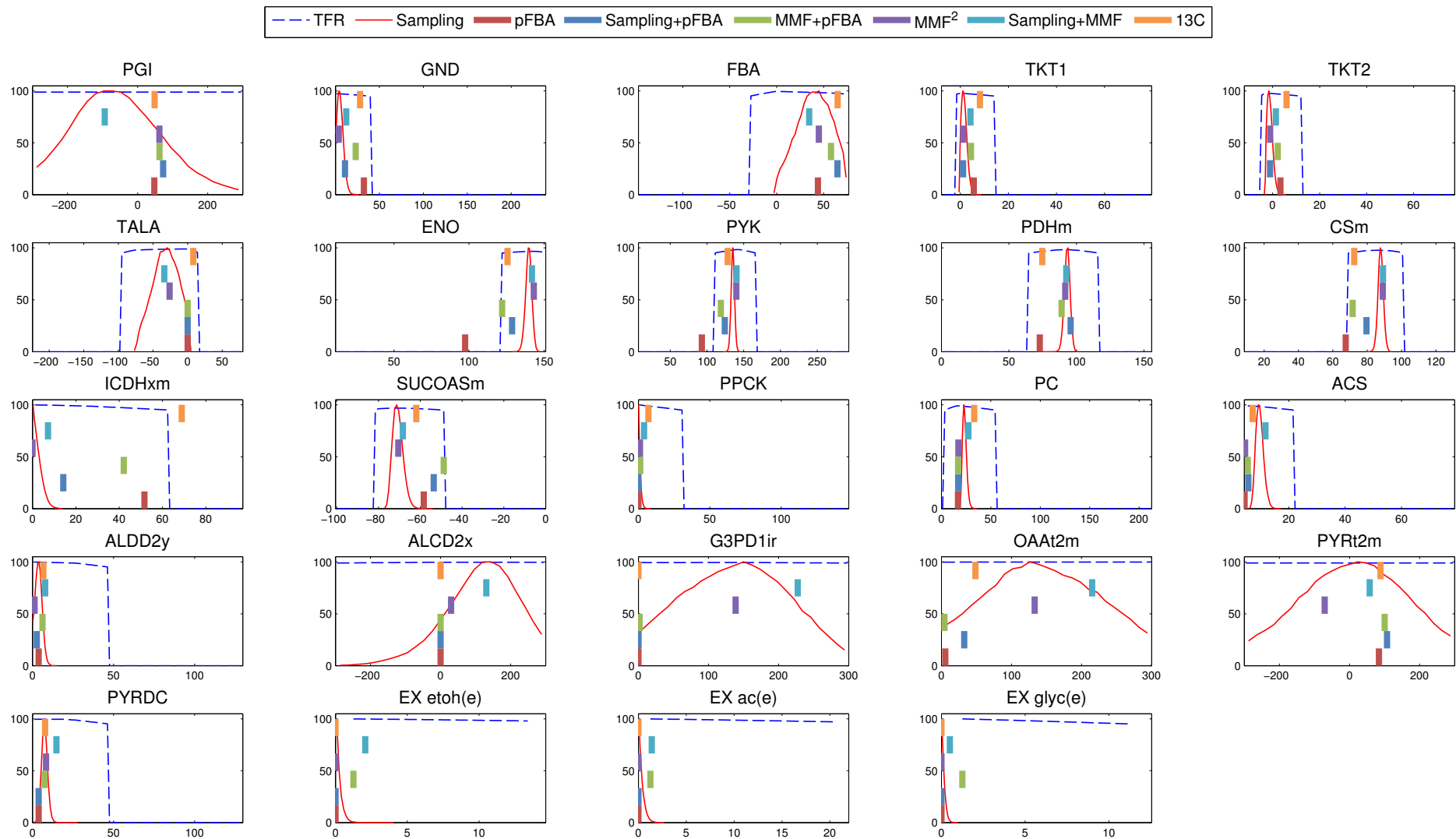

Figure 2

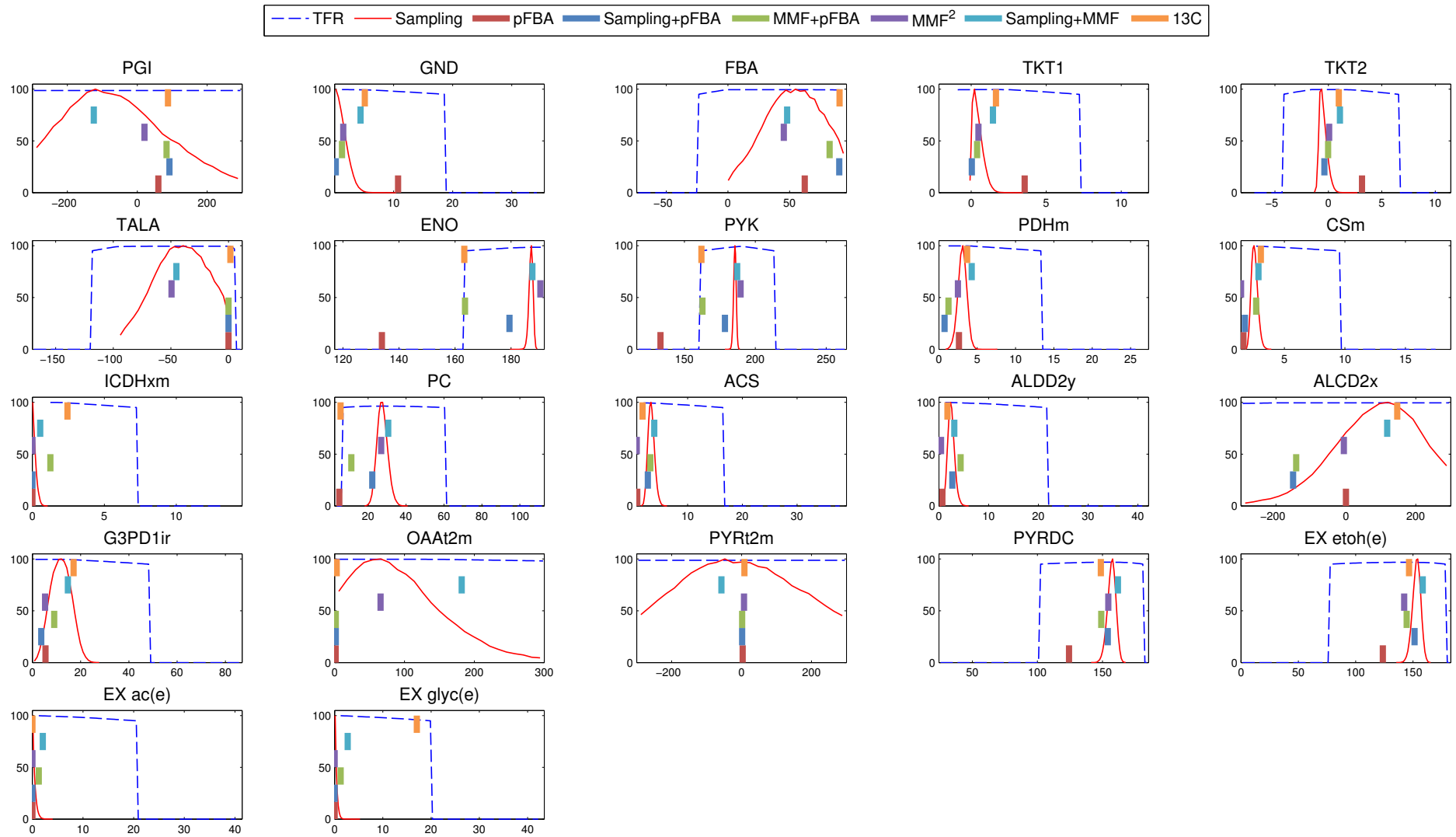

Figure 3

Supplement: S1 File — In this scenario, the glucose and oxygen consumption rates were set to their measured values, and the biomass flux was constrained to the observed growth rate. The TFR distribution depicts for each reaction the feasible rate that allows a TFR ≥ 0.95. Notice that most measured fluxes are indeed within the range that allows for this large “network flexibility”. The narrow sampling distributions allow for a larger reduction of the flux space, but unfortunately often do not capture the measured (13C) flux. Rectangular shapes indicate the predicted (pFBA and MMF variants) and measured flux (13C). E. coli iAF1260 (Holm et al.) network (Fig A). E. coli iAF1260 (Ishii et al.) network (Fig B). S. cerevisiae iMM904 high O2 (Rintala et al.) network (Fig C). S. cerevisiae iMM904 low O2 (Rintala et al.) network (Fig D). (PDF) [file pone.0139665.s001.pdf]

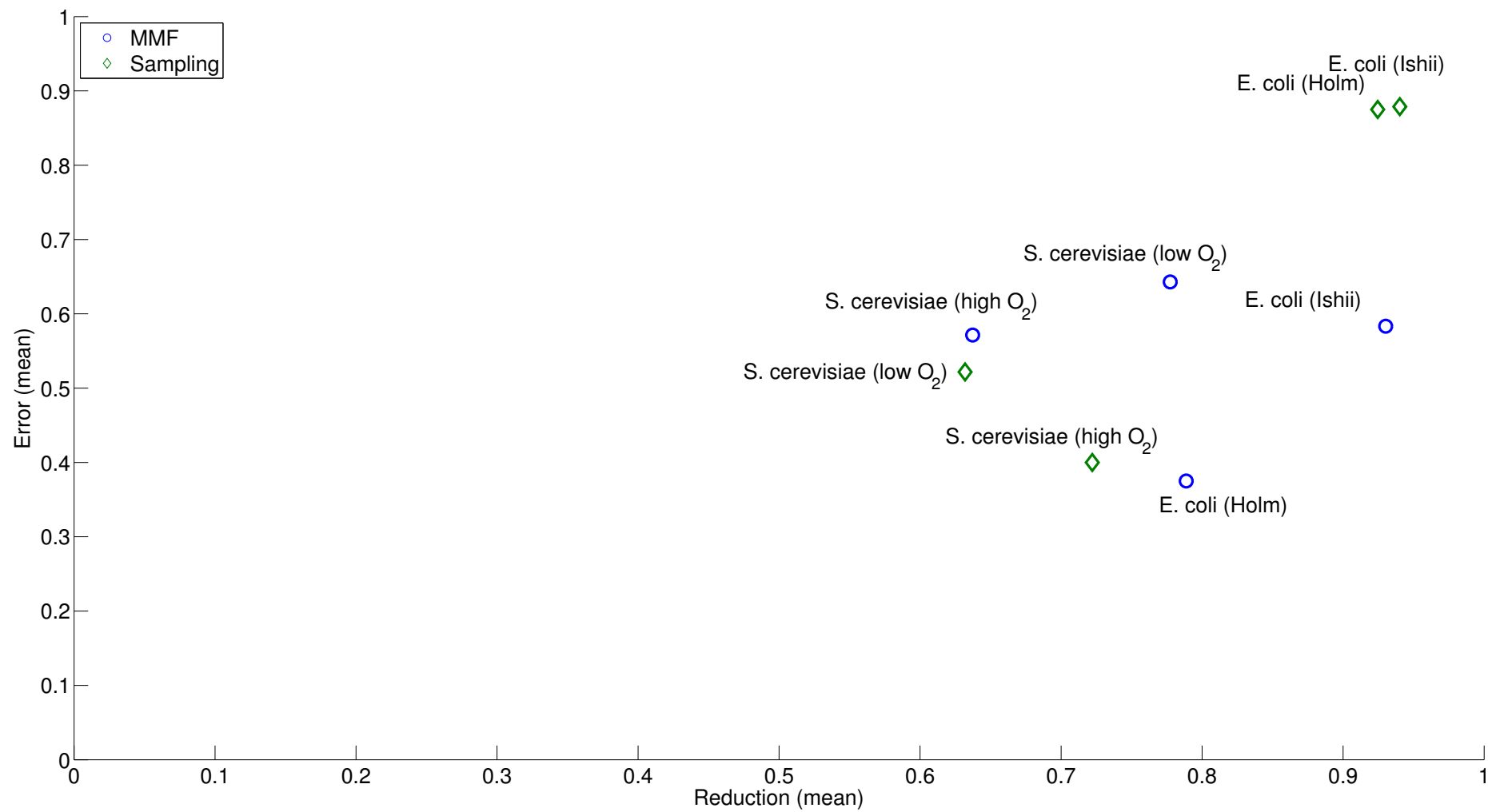

Figure 1

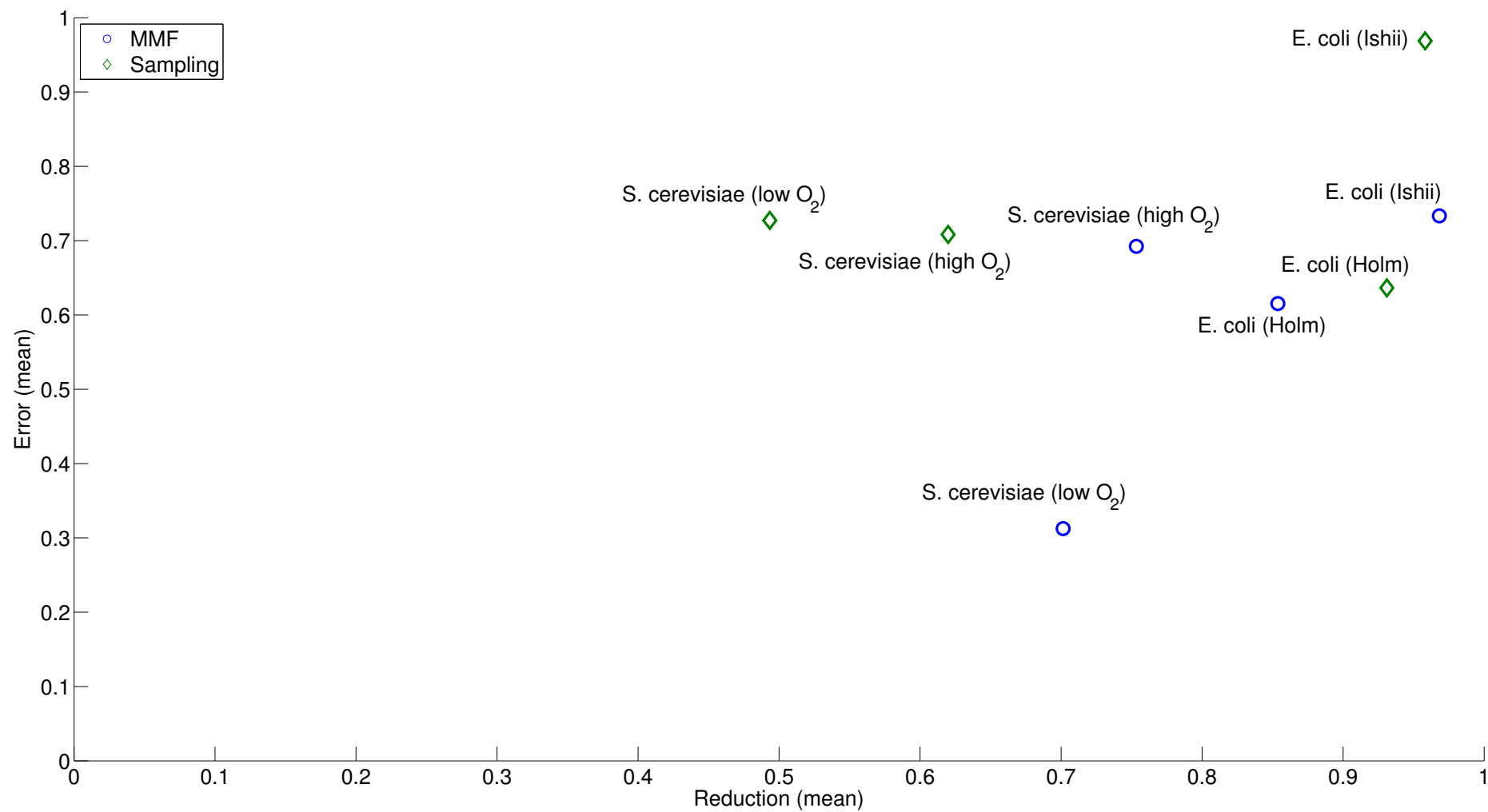

Figure 2

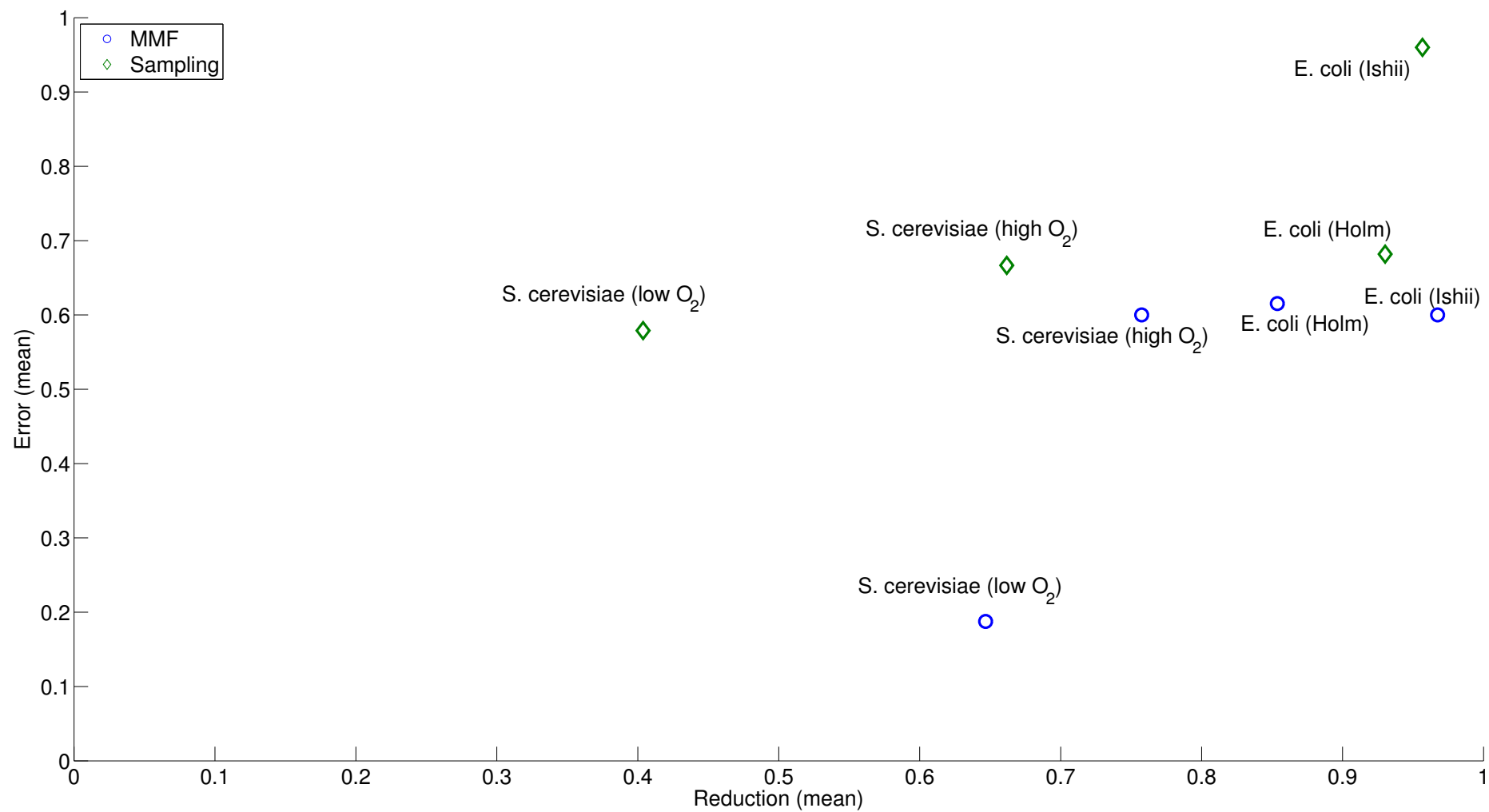

Figure 3

Supplement: S2 File — ACHR sampling compared to the MMF approach with TFR ≥ 0.99. Scenario 1: glucose and oxygen uptakes are constrained. MMF seems to perform better for the E. coli models compared to uniform sampling. For the yeast model with low oxygen, the reduction achieved by MMF is larger, but the error is also larger. Finally, uniform sampling performs better for the high oxygen yeast model. Notice that since the growth rate was not constrained, MMF in fact optimizes for “network flexibility” as its primary objective, which is probably incorrect (Fig A). Scenario 2: the uptake rates of glucose and oxygen, as well as the measured growth rate are set in the model. MMF performs better than random sampling for at least 3 of the 4 models (Fig B). Scenario 3: all exchange rates are constrained. Again MMF performs better on at least 3 of the 4 models (Fig C). (PDF) [file pone.0139665.s002.pdf]

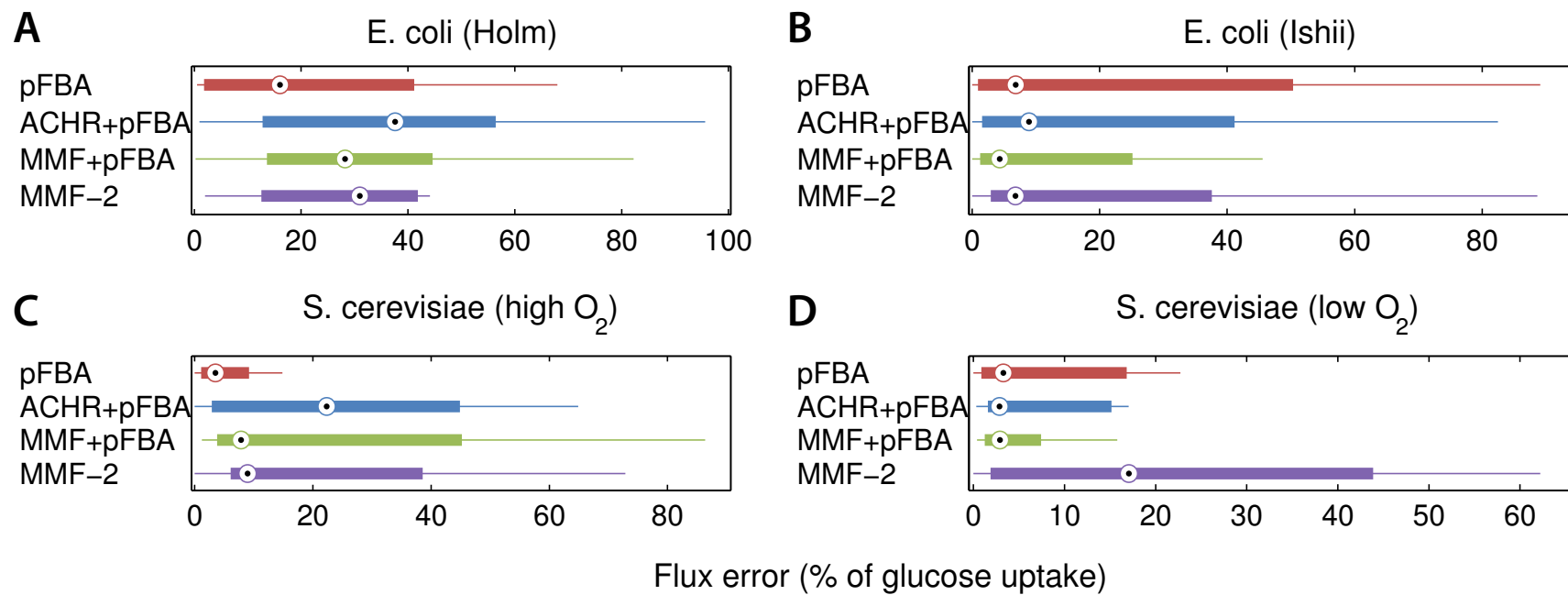

Figure 1

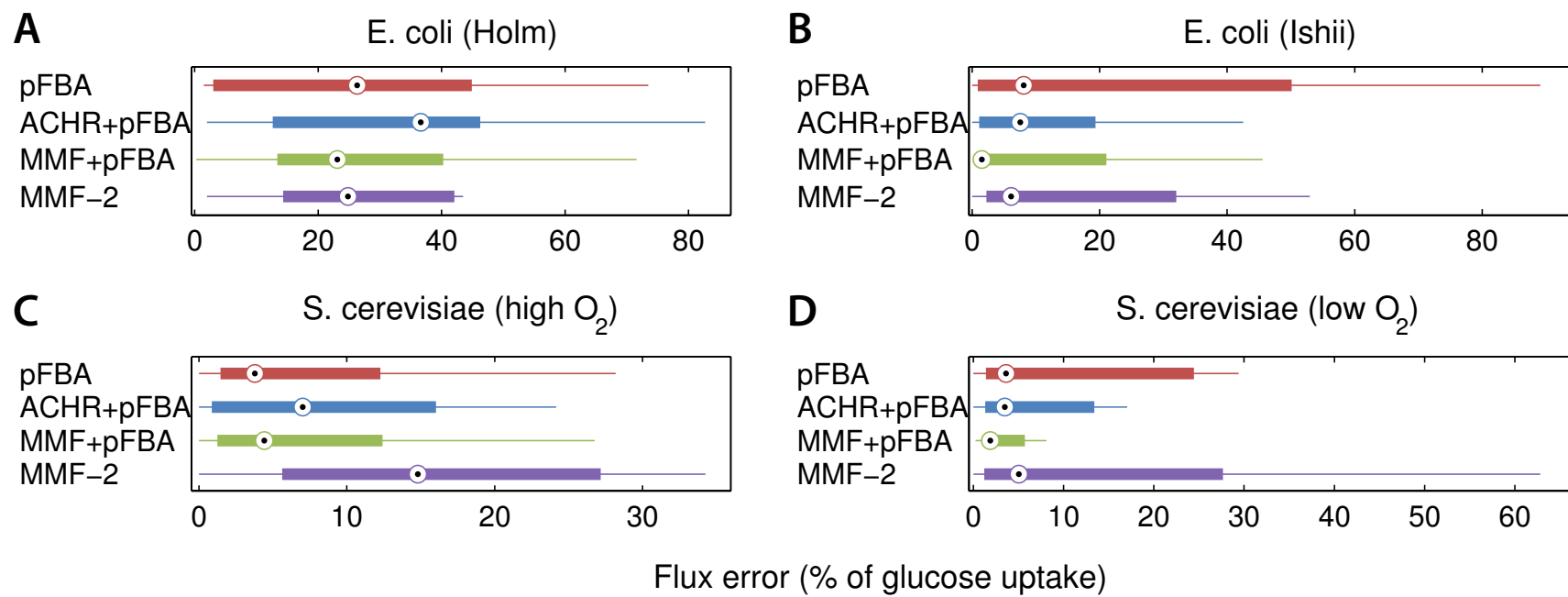

Figure 2

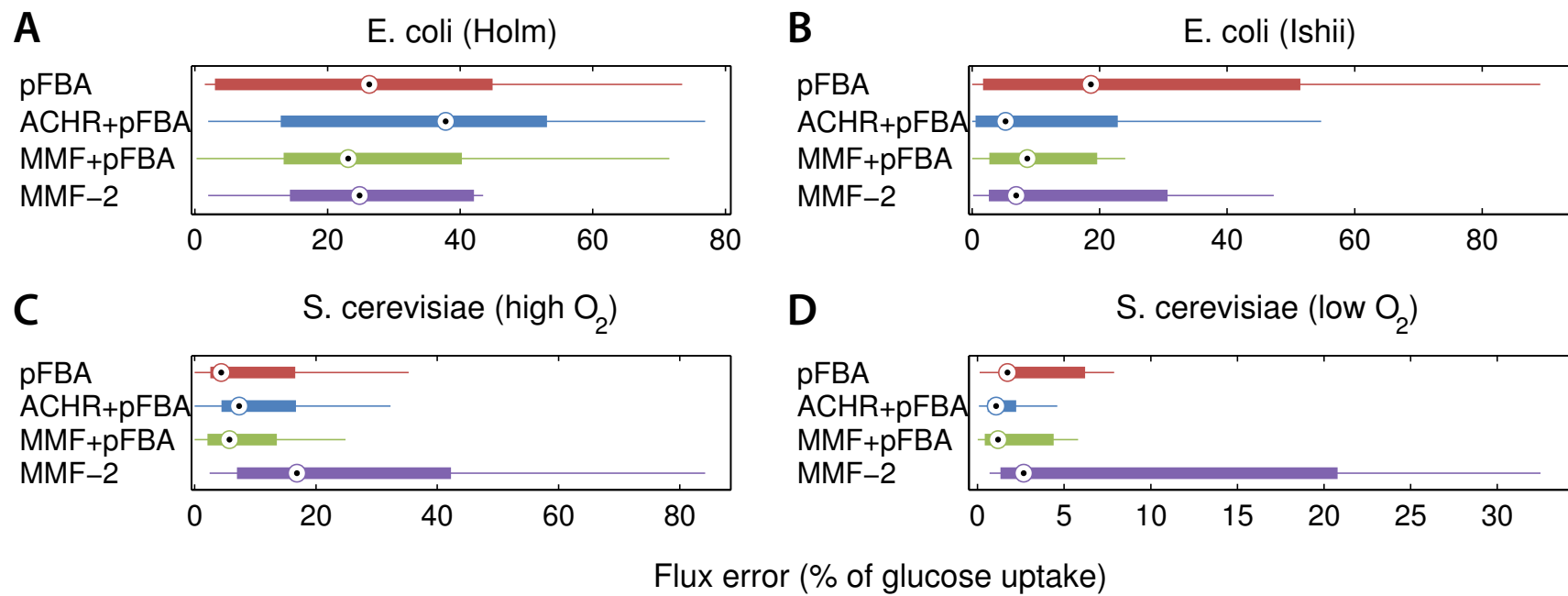

Figure 3

Supplement: S3 File — Scenario 1: constrained glucose and oxygen consumption. MMF does not consider biomass pressure, but is designed to optimize the network flexibility under sub optimal growth rates. Therefore, it does not perform well in this scenario, where the growth rate is unknown (Fig A). Scenario 2: the growth rate is constrained in addition to the glucose and oxygen consumption rates. In this scenario MMF performs better, because a sub optimal solution space is considered (Fig B). Scenario 3: all measured exchange fluxes are constrained. In this case, the MMF paradigm is also valid (Fig C). (PDF) [file pone.0139665.s003.pdf]
