# Supplementary material for: Estimating Metabolic Fluxes Using a Maximum Network Flexibility Paradigm
Source: PLoS One. 2015 Oct 12;10(10):e0139665. doi: 10.1371/journal.pone.0139665 (PMC4601694; doi:10.1371/journal.pone.0139665)
Supplement: S4 File — The grey line denotes the mean flux range in the reference model. Scenario 1: fixed glucose and oxygen uptake rates. In this setting, MMF provides the best reduction of the flux ranges for all models (Fig A). Scenario 2: the uptake rates of glucose and oxygen, as well as the measured growth rate are set in the model. Again MMF provides the best reduction of the flux ranges for all models (Fig B). Scenario 3: all exchange rates are constrained. Also in this case, the best overall TFR reduction is achieved by MMF (Fig C). (PDF) [file pone.0139665.s004.pdf]

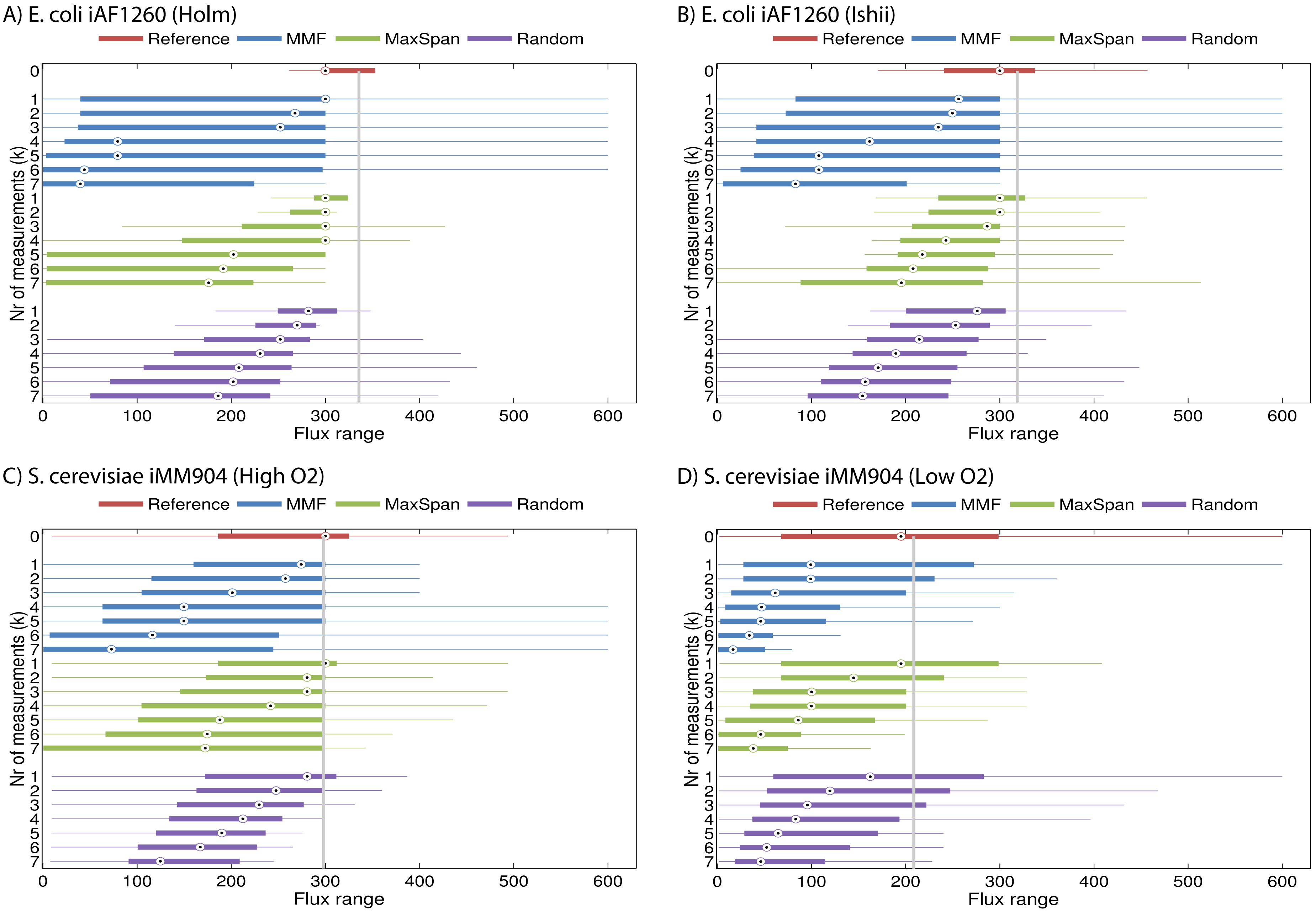
A) E. coli iAF1260 (Holm)
B) E. coli iAF1260 (Ishii)
C) S. cerevisiae iMM904 (High O2)
D) S. cerevisiae iMM904 (Low O2)
Reference
MMF
MaxSpan
Random
Nr of measurements (k)
Flux range
0
100
200
300
400
500
600
1
2
3
4
5
6
7

Figure 1

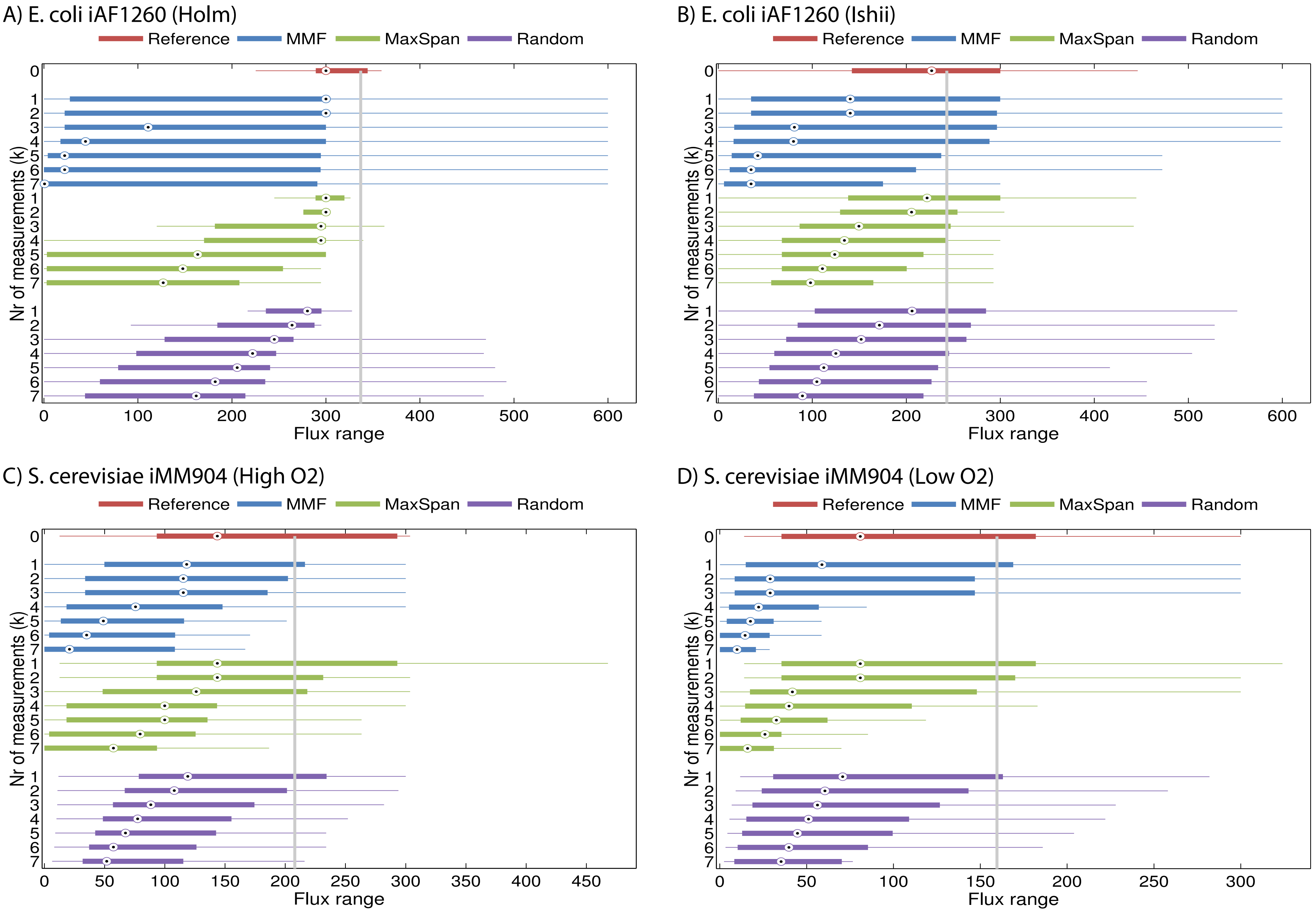
A) E. coli iAF1260 (Holm)
B) E. coli iAF1260 (Ishii)
C) S. cerevisiae iMM904 (High O2)
D) S. cerevisiae iMM904 (Low O2)
Reference
MMF
MaxSpan
Random
Nr of measurements (k)
Flux range

Figure 2

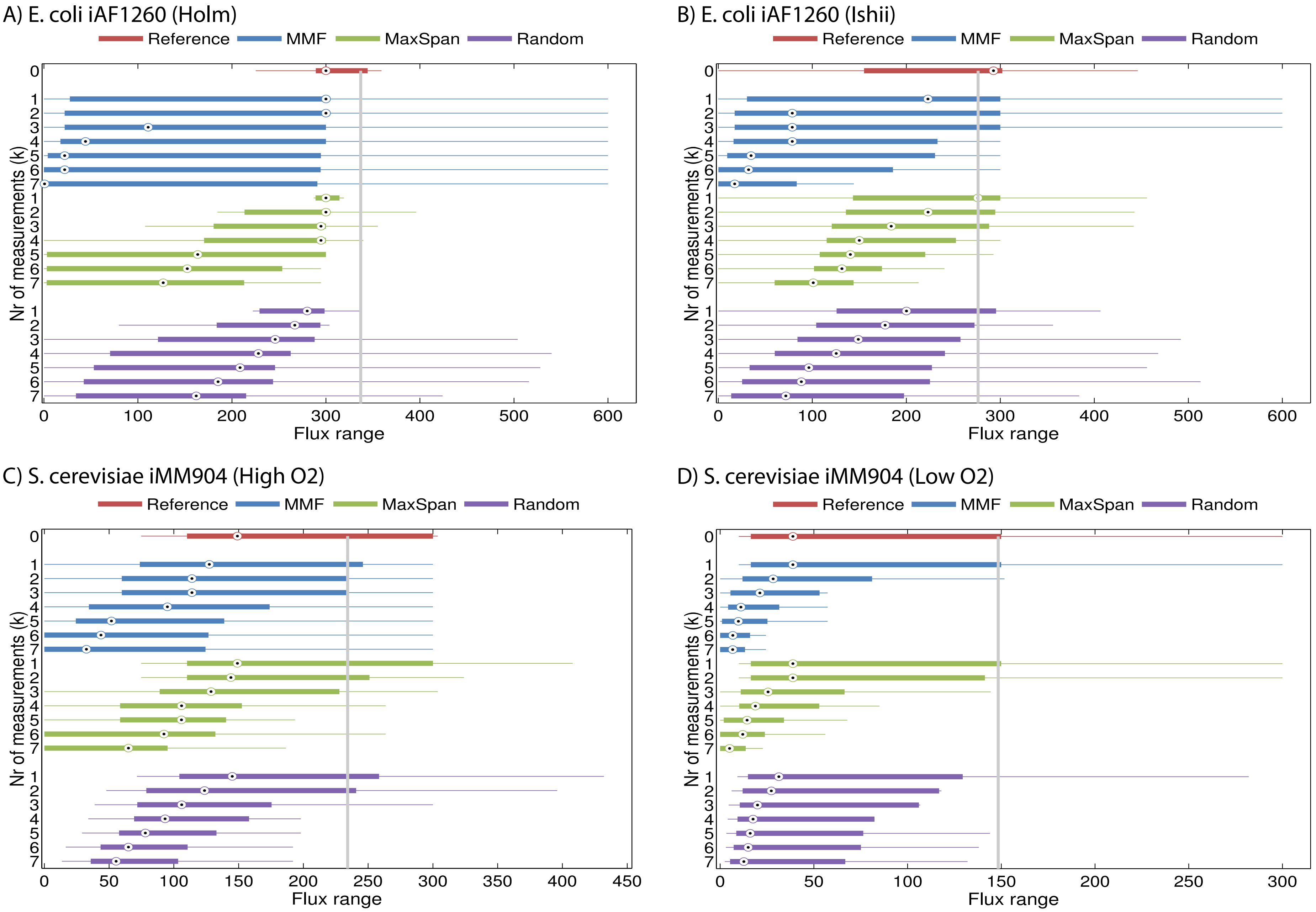
A) E. coli iAF1260 (Holm)
B) E. coli iAF1260 (Ishii)
C) S. cerevisiae iMM904 (High O2)
D) S. cerevisiae iMM904 (Low O2)
Reference
MMF
MaxSpan
Random
Nr of measurements (k)
Flux range

Figure 3
