# Supplementary material for: Estimating Metabolic Fluxes Using a Maximum Network Flexibility Paradigm
Source: PLoS One. 2015 Oct 12;10(10):e0139665. doi: 10.1371/journal.pone.0139665 (PMC4601694; doi:10.1371/journal.pone.0139665)
Supplement: S5 File — The grey line denotes the mean error in the reference model. Scenario 1: constrained glucose and oxygen rates. A smaller TFR does not necessarily correlate with a reduced pFBA error (Fig A). Scenario 2: the glucose and oxygen rates, as well as the biomass production rate are constrained. In most models, the strongly reduced prediction error correlates well with the reduced TFR, except for the E. coli iAF1260 (Ishii et al.) model (Fig B). Scenario 3: all exchange fluxes are constrained. Again, the error does not monotonically decrease with the number of constrained reactions, but does converge towards zero (Fig C). (PDF) [file pone.0139665.s005.pdf]

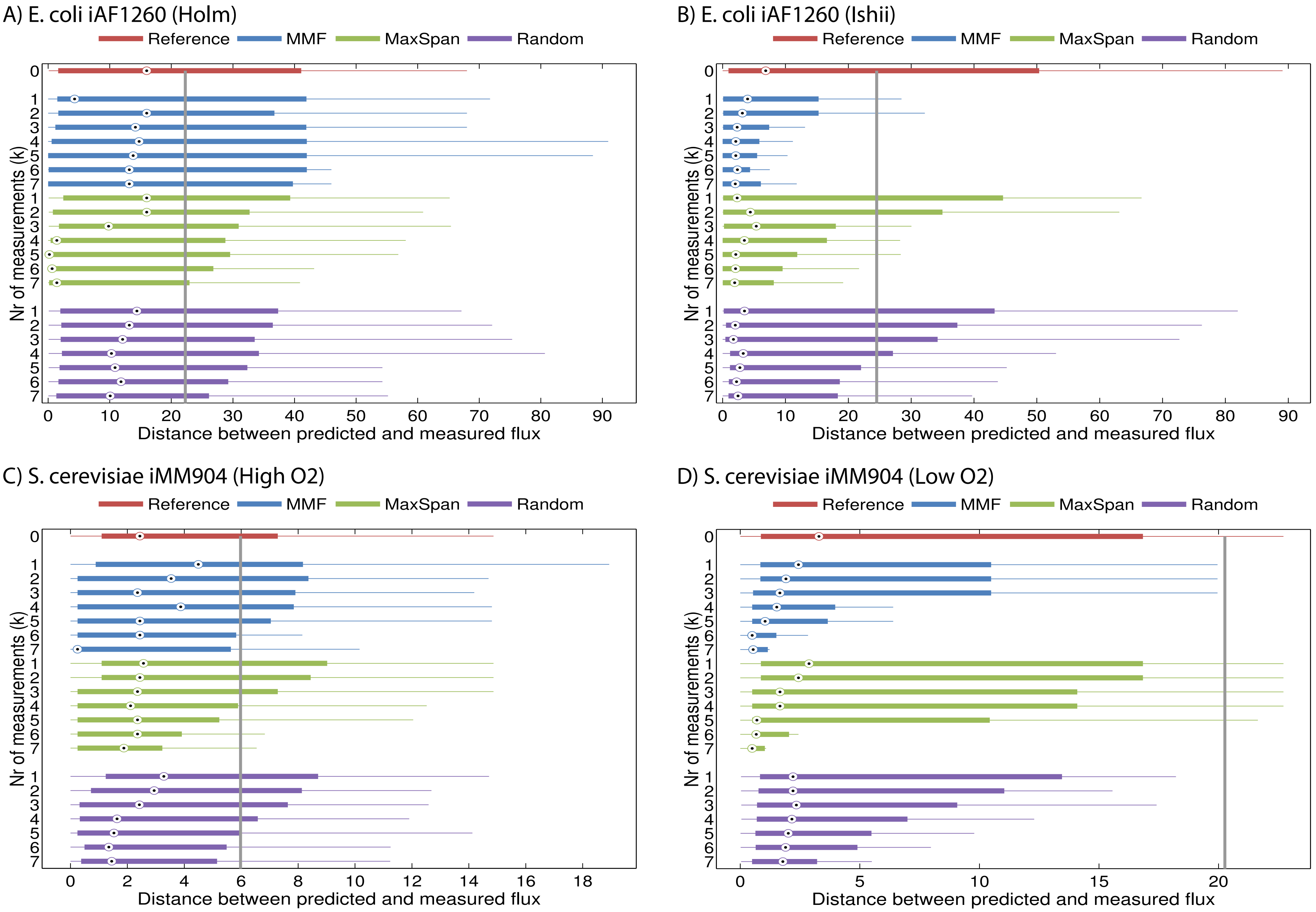
A) E. coli iAF1260 (Holm)
B) E. coli iAF1260 (Ishii)
C) S. cerevisiae iMM904 (High O2)
D) S. cerevisiae iMM904 (Low O2)
Reference
MMF
MaxSpan
Random
Nr of measurements (k)
Distance between predicted and measured flux

Figure 1

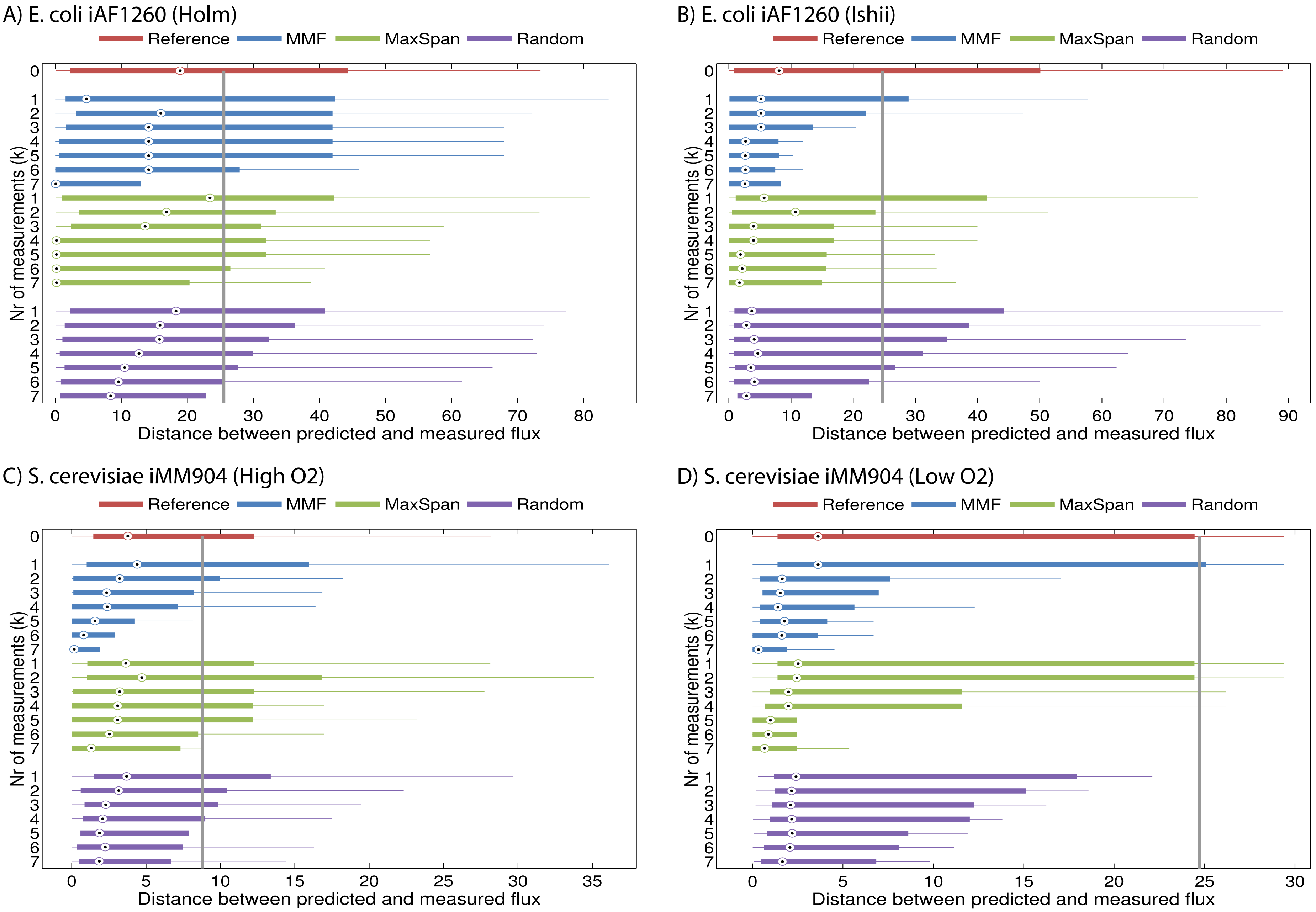

A) E. coli iAF1260 (Holm)
B) E. coli iAF1260 (Ishii)
C) S. cerevisiae iMM904 (High O2)
D) S. cerevisiae iMM904 (Low O2)
Reference
MMF
MaxSpan
Random
Nr of measurements (k)
Distance between predicted and measured flux

Figure 2

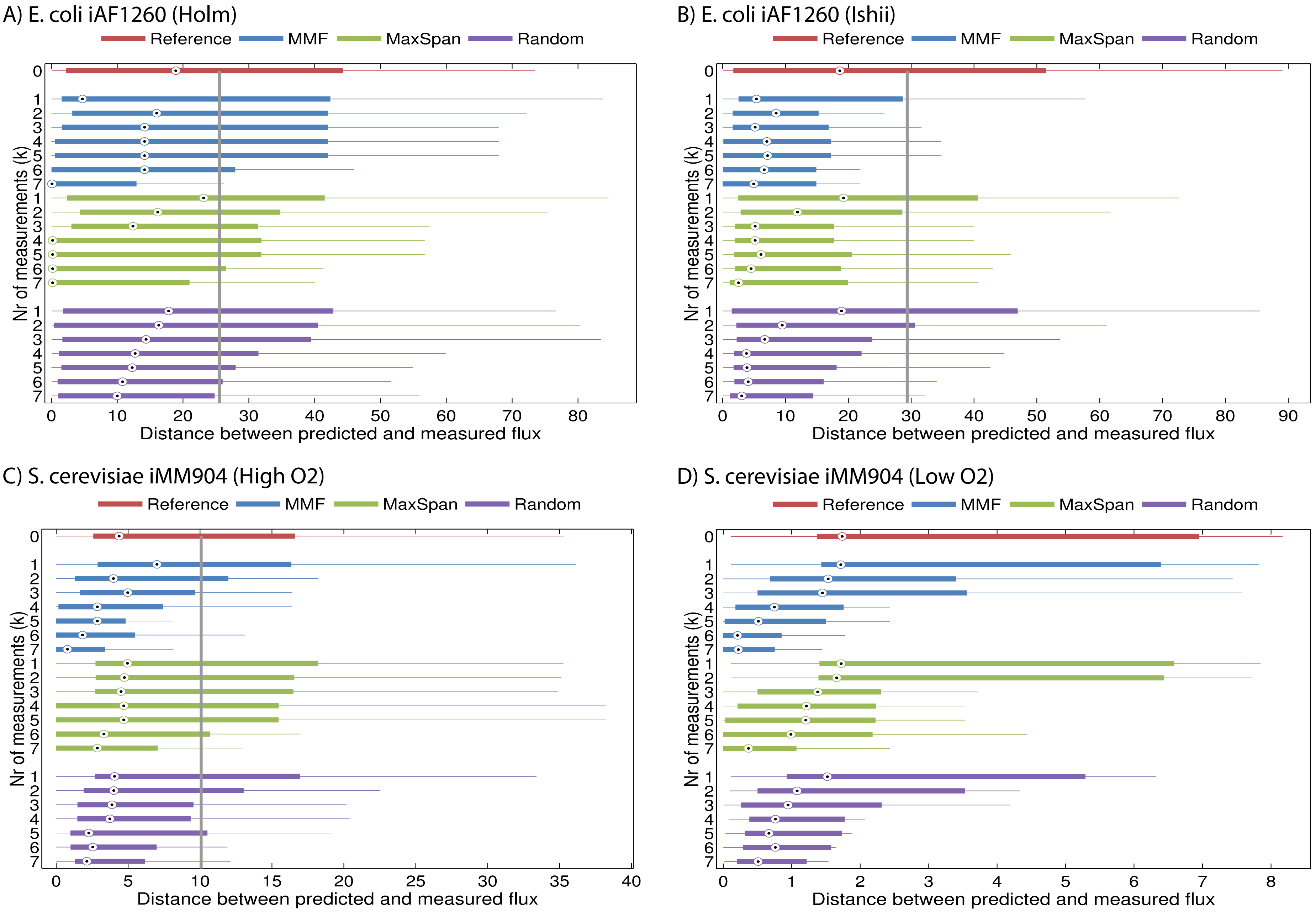
A) E. coli iAF1260 (Holm)
B) E. coli iAF1260 (Ishii)
C) S. cerevisiae iMM904 (High O2)
D) S. cerevisiae iMM904 (Low O2)
Reference
MMF
MaxSpan
Random
Nr of measurements (k)
Distance between predicted and measured flux

Figure 3
